# Type 2 Diabetes Mellitus: Molecular Pathogenesis and Therapeutic Interventions

**DOI:** 10.1002/mco2.70895

**Published:** 2026-08-03

**Authors:** Shinuan Fei, Xiyue Xiong, Lu Zhang, Jian Zhang, Lawrence W. C. Chan, Hong Jiang, Qingqing Xiong, Changtao Hu, Jie Chen, Sijun Yang

**Affiliations:** ^1^ Pediatrics Department Huangshi Maternal and Child Health Care Hospital Affiliated Huangshi Maternal and Child Health Care Hospital of Hubei Polytechnic University Huangshi Key Laboratory of Birth Defects Prevention Huangshi Hubei China; ^2^ Nanjing University of Information Science and Technology Nanjing Jiangsu China; ^3^ School of Design Huazhong University of Science and Technology Wuhan Hubei China; ^4^ Key Laboratory of Lighting Interactive Service and Technology Ministry of Culture and Tourism (MoCT Key Laboratory of Lighting Interactive Service&Tech) Hubei Engineering Research Center for Tech of Digital Lighting School of Design Huazhong University of Science and Technology Wuhan Hubei China; ^5^ Department of Health Technology and Informatics Hong Kong Polytechnic University Hong Kong Hong Kong China; ^6^ Department of Laboratory Medicine Huangshi Maternal and Child Health Care Hospital Affiliated Huangshi Maternal and Child Health Care Hospital of Hubei Polytechnic University Huangshi Key Laboratory of Birth Defects Prevention Huangshi China; ^7^ Huangshi Key Laboratory of Birth Defects Prevention Huangshi Maternal and Child Health Care Hospital Affiliated Huangshi Maternal and Child Health Care Hospital of Hubei Polytechnic University Huangshi Key Laboratory of Birth Defects Prevention Huangshi Hubei China; ^8^ Department of Respiratory Medicine Huangshi Maternal and Child Health Care Hospital Affiliated Huangshi Maternal and Child Health Care Hospital of Hubei Polytechnic University Huangshi Key Laboratory of Birth Defects Prevention Huangshi Hubei China; ^9^ Hubei Provincial Key Laboratory of Occurrence and Intervention of Kidney Diseases School of Medicine Hubei Polytechnic University Huangshi Hubei China

**Keywords:** β‐cell dysfunction, mitochondrial dynamics, molecular pathogenesis, therapeutic interventions, Type 2 diabetes

## Abstract

Type 2 diabetes mellitus (T2D) is a pervasive metabolic disorder driven by insulin resistance and progressive pancreatic β‐cell failure, with a rapidly growing global prevalence. Its pathogenesis now extends beyond hyperglycemia to encompass intricate immunometabolic dysregulation, mitochondrial dysfunction, and organelle stress. However, a cohesive synthesis that integrates these disparate molecular mechanisms with contemporary therapeutic advancements remains missing. This review systematically investigates the immunometabolic axis, highlighting macrophage polarization and exosomes, as well as nanotube‐mediated crosstalk with β‐cells, and describes how mitochondrial dynamics disorder and impaired mitophagy become the core driving factors for β‐cell failure. Lipotoxicity mediated by ceramides, diacylglycerols, and cholesterol imbalance is critically analyzed alongside proteotoxicity from islet amyloid polypeptide aggregation. We then chart the therapeutic evolution from glucocentric strategies to modern complication‐centric paradigms, emphasizing SGLT2 inhibitors and GLP‐1 receptor agonists that confer proven cardio‐renal protection. Personalized treatment, multiomics integration, next‐generation precision therapies, and holistic art‐based interventions that promote sustainable lifestyle changes are further discussed. By connecting molecular insights to clinical application, this review provides a comprehensive resource for researchers and clinicians, aiming to advance T2D management and improve global patient outcomes.

## Graphical abstract

This graphical summary illustrates the multiorgan therapeutic landscape for Type 2 diabetes mellitus (T2D), integrating molecular pathogenesis with modern treatment strategies. It depicts how current interventions, including SGLT2 inhibitors, GLP‐1 receptor agonists, and insulin secretagogues, target key organs—kidney, pancreas, gastrointestinal tract, adipose tissue, and central nervous system—to correct hyperglycemia, insulin resistance, and β‐cell dysfunction. The figure underscores the paradigm shift from glucocentric management to a complication‐centric approach, emphasizing organ protection and personalized therapy to address the underlying metabolic dysregulation of T2D.

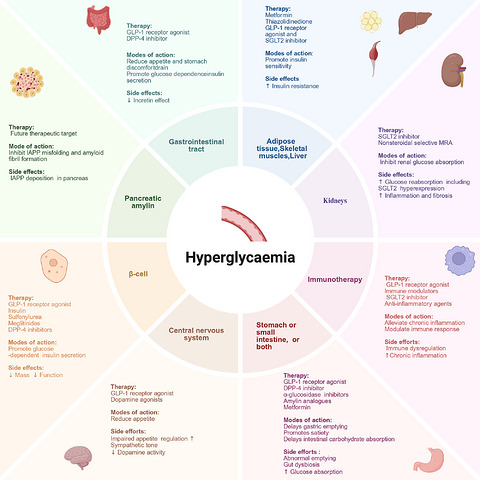

## 1 Introduction

Type 2 diabetes (T2D) represents a pervasive global health challenge, with its prevalence projected to rise dramatically from 529 million to 1.3 billion cases by 2050 [1]. It is now recognized as a complex metabolic syndrome characterized by a vicious cycle of insulin resistance (IR) in peripheral tissues and the progressive failure of pancreatic β‐cells [2]. The pathogenesis is no longer attributed to a single defect but is understood to involve a complex interplay of genetic predisposition, epigenetic modifications, and environmental factors such as obesity and sedentary lifestyles [2]. Current research has moved beyond a glucocentric perspective, increasingly focusing on the roles of chronic low‐grade inflammation, mitochondrial dysfunction, and organelle stress in driving the disease's progression and its devastating complications, including cardiovascular disease (CVD), nephropathy, and neuropathy.

The impetus for writing this review stems from the rapid expansion of knowledge in T2D pathogenesis and the consequent paradigm shift in therapeutic strategies. While existing literature often delves into specific pathways, there is a pressing need for a comprehensive synthesis that connects the dots between disparate mechanisms such as immunometabolism, mitochondrial dynamics, and lipotoxicity, and links them directly to the latest advancements in treatment. This review aims to bridge this gap by providing an integrated overview of how these molecular pathways converge to drive β‐cell dysfunction and systemic metabolic dysregulation. We critically assess how emerging concepts, like macrophage plasticity and β‐cell dedifferentiation, are reshaping our understanding of the disease and opening new avenues for intervention, justifying the need for an up‐to‐date, holistic resource for researchers and clinicians.

The main content and highlights of this review are multifaceted. A primary highlight is the in‐depth exploration of the immunometabolic axis, particularly moving beyond the traditional proinflammatory (M1)/anti‐inflammatory (M2) macrophage dichotomy to discuss a spectrum of polarization states and their sophisticated crosstalk with β‐cells via exosomes and tunneling nanotubes (TNTs). Another significant focus is on mitochondrial mechanisms, where we detail how disrupted dynamics (fusion/fission) and impaired quality control (mitophagy) are central to β‐cell failure. Furthermore, the review provides a critical analysis of lipotoxicity, examining the distinct roles of ceramides, diacylglycerols (DAGs), and disrupted cholesterol homeostasis in promoting IR and β‐cell apoptosis. A particularly innovative highlight is the incorporation of art‐based interventions, which serve as a bridge between psychological support and community‐wide health engagement, fostering sustainable behavioral change and reducing emotional barriers to diabetes management. Finally, a key highlight is the transition from describing pathogenesis to reviewing the evolution of therapeutic interventions, from traditional glucose‐lowering agents to modern drugs with cardio‐renal benefits and emerging strategies targeting underlying disease mechanisms.

This review is structured to provide a logical progression from molecular mechanisms to clinical applications. We begin by exploring core pathological pathways, including the immunometabolic axis and mitochondrial dysfunction in β‐cells, followed by an in‐depth analysis of key signaling cascades such as insulin signaling, inflammatory pathways, ER stress, and oxidative stress. Subsequent sections address β‐cell dysfunction, encompassing dedifferentiation, senescence, and secretory defects, and the roles of ectopic lipid deposition and proteotoxicity. We also examine neuroendocrine dysregulation and the emerging potential of art‐based and community‐driven interventions. The review culminates in a comprehensive discussion of therapeutic strategies, spanning lifestyle modifications, pharmacological advances, and personalized management approaches. This sequential organization ensures a coherent and systematic journey from molecular insights to practical clinical implications, offering a holistic perspective on T2D pathogenesis and management.

## 2 Molecular Pathogenesis of T2D

**FIGURE 1:**
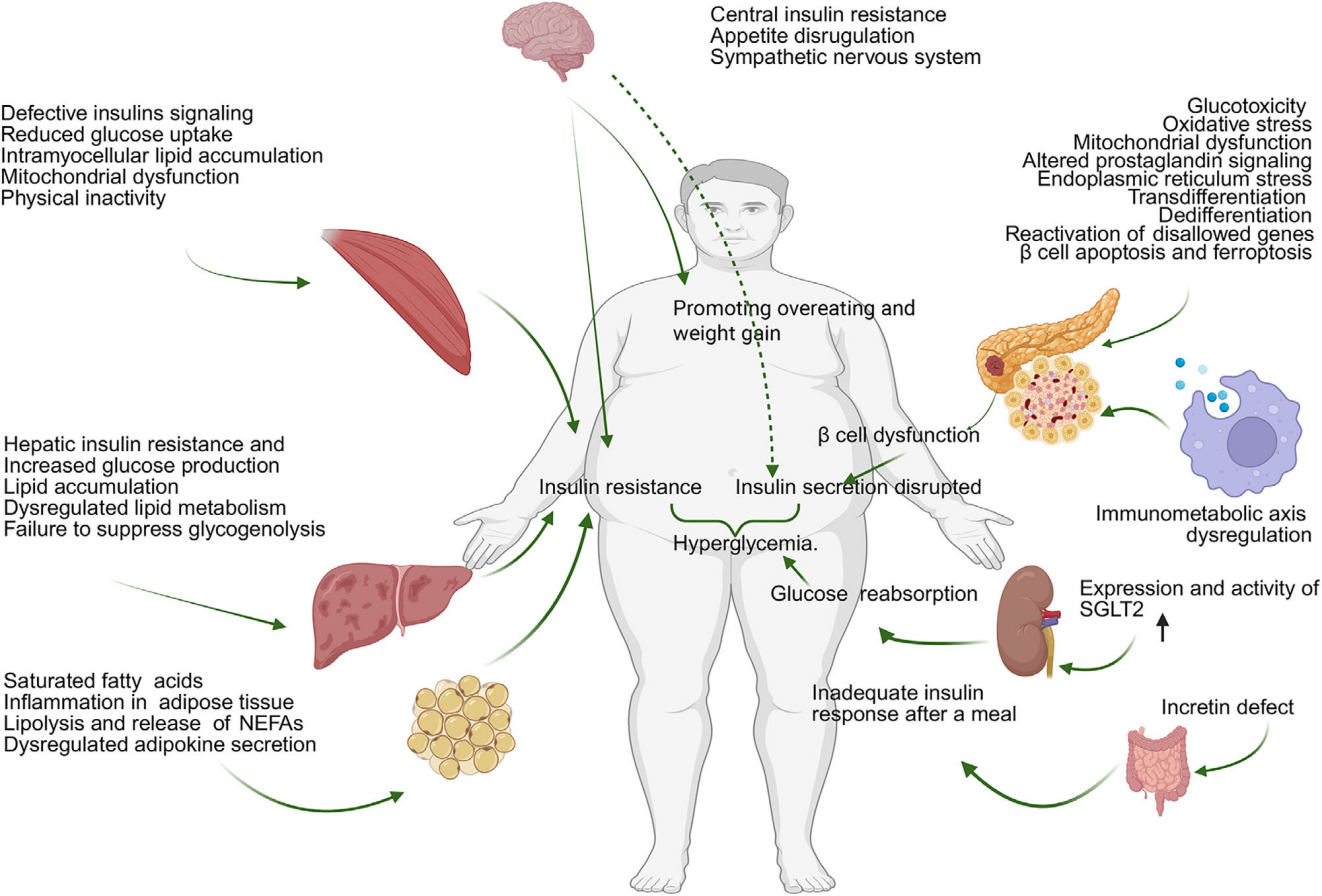
The pathogenesis of T2D. Based on the detailed diagram, the pathogenesis of T2D unfolds as a vicious cycle of metabolic dysfunction, primarily driven by the interplay between IR and progressive β‐cell failure. The cycle often originates with insulin resistance in skeletal muscle and liver tissue. In muscle, defective insulin signaling, exacerbated by intramyocellular lipid accumulation and physical inactivity, leads to reduced glucose uptake. Simultaneously, hepatic insulin resistance results in uncontrolled glucose production through increased gluconeogenesis and failure to suppress glycogenolysis. This dual defect creates a state of persistent hyperglycemia. Compounding this, dysfunctional adipose tissue releases saturated fatty acids and proinflammatory adipokines, which further intensify insulin resistance in other tissues. Initially, pancreatic β‐cells compensate by hypersecreting insulin. However, chronic exposure to this toxic environment of hyperglycemia (glucotoxicity) and elevated free fatty acids (lipotoxicity) triggers oxidative stress, endoplasmic reticulum stress, and inflammatory pathways within the β‐cells. This leads to their dedifferentiation, apoptosis, and ultimately, a critical decline in insulin secretory capacity. The resulting inadequate insulin response post‐meal solidifies the hyperglycemic state. This hyperglycemia is then maintained by several positive feedback loops: it directly accelerates β‐cell failure, and through upregulation of SGLT2 transporters in the kidney, it increases glucose reabsorption, trapping glucose in the bloodstream. This chronic, low‐grade inflammatory state is critically shaped by the polarization of macrophages toward a proinflammatory (M1) phenotype within metabolic tissues. These M1 macrophages amplify tissue inflammation and IR, while simultaneously impairing pancreatic β‐cell function, thereby forging a key link between inflammation and metabolic demise. Furthermore, central insulin resistance in the brain disrupts appetite regulation, promoting overeating and weight gain, which in turn fuels the underlying adipose tissue dysfunction and insulin resistance. Thus, T2D is a self‐perpetuating disorder where defects in muscle, liver, fat, pancreas, kidney, and brain converge to create and sustain a pathological hyperglycemic state. T2D, Type 2 diabetes; IR, insulin resistance; SGLT2, sodium–glucose cotransporter‐2.

While the vicious cycle of IR and β‐cell failure frames our understanding of T2D clinically, a critical question remains: what are the underlying molecular insults that initiate and perpetuate this cycle? The answer lies in a complex interplay of cellular events, beginning with a dysregulated immunometabolic axis where macrophage polarization orchestrates local inflammation, and extending to the very energy centers of the β‐cell, where mitochondrial dynamics and quality control become critically impaired. Furthermore, this pathogenic network extends to encompass lipotoxicity from specific lipid species, proteostasis imbalance, and dysregulation across multiple key signaling pathways, collectively driving the vicious cycle of IR and β‐cell failure. For a schematic overview of the pathogenesis of T2D, refer to Figure 1.

### 2.1 Immunometabolic Axis: The Central Role of Macrophage Polarization in T2D

As a complex metabolic disorder, T2D involves intricate interactions across multiple tissues and cell types. Among these, macrophage polarization emerges as a pivotal immunometabolic axis, critically shaping both systemic IR and pancreatic β‐cell dysfunction. It moves beyond the traditional M1/M2 dichotomy to explore the continuum of macrophage phenotypes and their sophisticated crosstalk with β‐cells [3].

#### 2.1.1 Beyond M1/M2: A Spectrum of Macrophage Phenotypes in T2D

**FIGURE 2:**
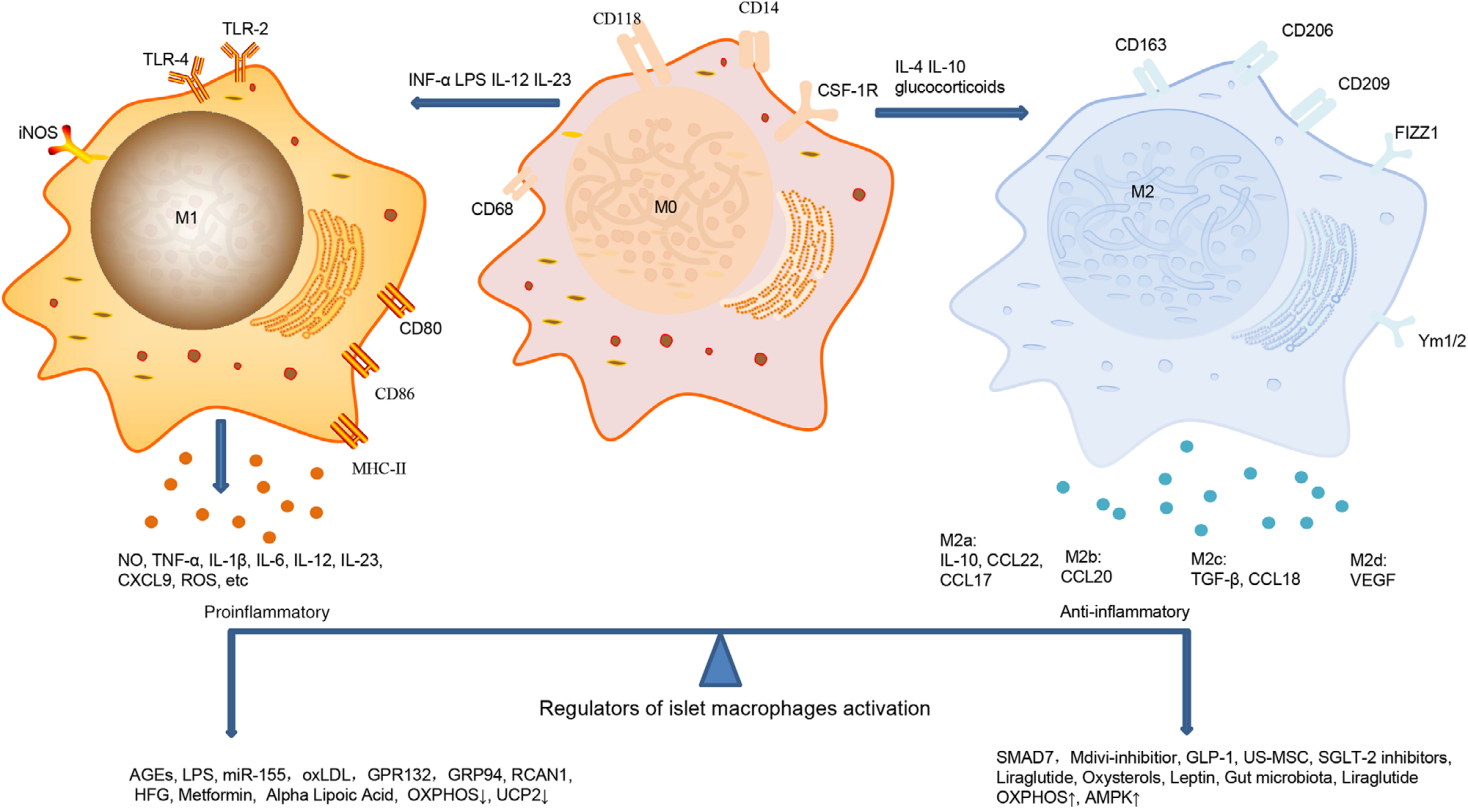
Regulators of islet macrophages activation. Quiescent macrophages (M0) possess the plasticity to polarize into two distinct phenotypes—proinflammatory M1 and anti‐inflammatory M2, depending on microenvironmental cues. M1 macrophages are activated by proinflammatory stimuli such as LPS, TNF‐α, IFN‐γ, miR‐155, IL‐12, and IL‐23, as well as metabolic stressors like AGEs, oxLDL, and high‐fat high‐glucose (HFG) conditions. They express characteristic surface markers including TLR2, TLR4, MHC‐II, CD86, CD80, CD68, and CD14. These cells exhibit a glycolytic metabolic profile characterized by decreased oxidative phosphorylation (OXPHOS) and reduced uncoupling protein 2 (UCP2) expression, driving the production of inflammatory mediators including NO, TNF‐α, IL‐1β, IL‐6, and ROS. Therapeutic strategies to suppress M1 polarization include metformin and alpha‐lipoic acid. In contrast, M2 macrophages are activated by anti‐inflammatory signals such as IL‐4, IL‐10, IL‐13, glucocorticoids, and metabolites from gut microbiota. They display a reparative phenotype marked by OXPHOS‐dependent fatty acid oxidation and enhanced AMPK activity, secreting anti‐inflammatory factors like IL‐10, TGF‐β, and VEGF while expressing surface markers CD206, CD163, CD209, FIZZ1, and Ym1/2. Pharmacological interventions such as Mdivi‐1 (a mitochondrial fusion promoter) and liraglutide (a GLP‐1 agonist) enhance M2 polarization by stabilizing mitochondrial dynamics and amplifying anti‐inflammatory signaling. Additionally, emerging therapies like SGLT‐2 inhibitors and ultrasound‐stimulated mesenchymal stem cells (US‐MSCs) further reinforce the M2 phenotype through metabolic reprogramming and niche modulation. This dichotomy underscores the dual role of macrophages in T2D progression, where balancing M1/M2 polarization through metabolic and immunomodulatory interventions offers promising avenues for restoring β‐cell function and insulin sensitivity. M0, quiescent macrophages; M1, proinflammatory macrophages; M2, anti‐inflammatory macrophages; miR‐155, microRNA‐155; AGEs, advanced glycation end‐products; oxLDL, oxidized low‐density lipoprotein; HFG, high‐fat high‐glucose; TLR2, Toll‐like receptor 2; TLR4, Toll‐like receptor 4; MHC‐II, major histocompatibility complex Class II; CD86, cluster of differentiation 86; CD80, cluster of differentiation 80; CD68, cluster of differentiation 68; CD14, cluster of differentiation 14; CD206, cluster of differentiation 206; CD163, cluster of differentiation 163; CD209, cluster of differentiation 209; UCP2, uncoupling protein 2; TGF‐β, transforming growth factor‐beta; VEGF, vascular endothelial growth factor; FIZZ1, found in inflammatory zone 1; Ym1/2, chitinase‐like protein 3; AMPK, AMP‐activated protein kinase; US‐MSCs, ultrasound‐stimulated mesenchymal stem cells.

The traditional M1/M2 dichotomy, while foundational, is insufficient to capture the complexity of macrophage biology in T2D, as evidenced by in vivo studies showing a continuum of macrophage phenotypes that includes M1‐like, M2‐like, and hybrid phenotypes [4]. Single‐cell RNA sequencing has unveiled a spectrum of distinct macrophage subsets within metabolic tissues, each with unique transcriptional signatures and functional roles. For instance, MHC‐IIhi macrophages, prevalent in lean states, support β‐cell proliferation via IGF‐1, thereby preserving β‐cell mass and function, as demonstrated by the identification of the IGF‐1–IGF1R axis contributing to an anergic‐like T cell phenotype in islets [5]. In contrast, Ccr2hi macrophages drive inflammation and β‐cell apoptosis through the CXCL10–CXCR3 axis, contributing to IR, which aligns with findings that recruited macrophages play neuroprotective roles in peripheral nerves of obese prediabetic mice [6]. A disease‐specific RELAhigh cluster, identified in human diabetic islets, is characterized by hyperactivated NF‐κB signaling, elevated IL‐1β secretion, and a strong correlation with β‐cell failure, consistent with observations of macrophage subsets orchestrating tissue‐specific functions during homeostasis and disease [7]. Another subset, Rarres2^+^ macrophages in adipose tissue, alleviates IR by modulating adipokine expression via the Pla2g2d–GPR120 pathway, reflecting the unique metabolic functions of adipose tissue macrophages distinct from those in other tissues [7]. These discoveries underscore the need to move beyond the binary M1/M2 model and adopt a continuum‐based understanding of macrophage plasticity in T2D, as highlighted by recent advancements in characterizing tissue‐specific macrophage subpopulations with distinct developmental trajectories and transcriptional programs [4]. Quiescent macrophages (M0) can polarize into proinflammatory M1 or anti‐inflammatory M2 phenotypes depending on the local microenvironmental cues (Figure 2).

#### 2.1.2 Mechanisms of Macrophage–β‐Cell Crosstalk: Contact, Exosomes, and Nanotubes

**FIGURE 3:**
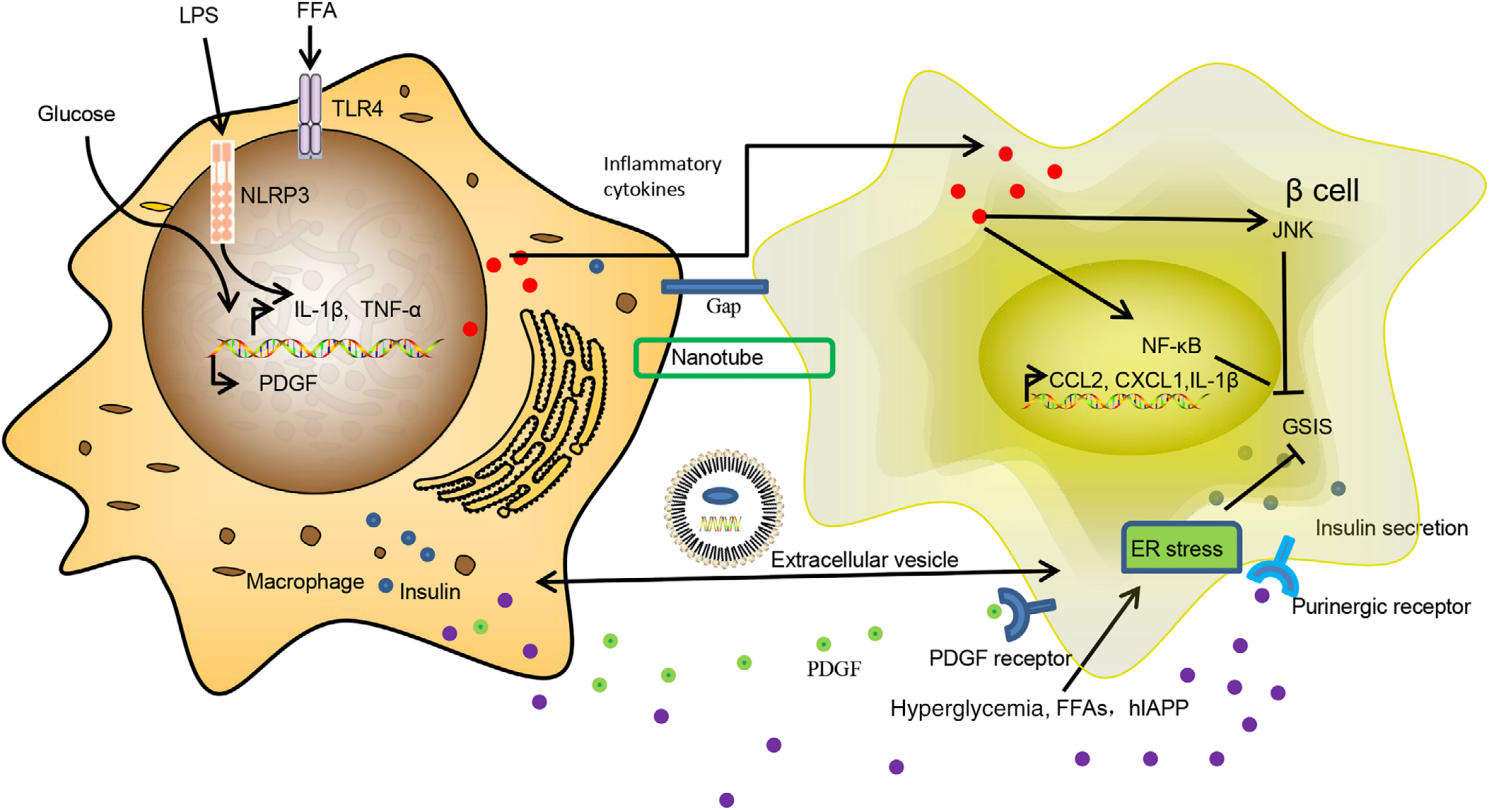
Multimodal crosstalk between proinflammatory macrophages and β‐cells in T2D pathogenesis. In the context of T2D, β‐cells attempt to counteract excessive nutrition and high blood glucose levels by secreting additional insulin. Macrophages, in turn, detect these alterations in β‐cell activity through the internalization of insulin‐containing vesicles and the heightened activity of purinergic receptors. The presence of increased glucose and FFAs in the blood can trigger islet macrophages to adopt a proinflammatory stance. Consequently, these macrophages generate larger quantities of inflammatory mediators, including IL‐1β and TNF, which then initiate signaling cascades such as NF‐κB and JNK within β‐cells, further aggravating ER stress. Collectively, these processes impair the ability of β‐cells to secrete insulin in response to glucose stimulation (GSIS). Beyond the effects of inflammatory cytokines, there are additional pathways through which macrophages may contribute to β‐cell dysfunction. These include the transfer of insulin via extracellular vesicles released by β‐cells and ingested by islet macrophages, as well as the establishment of tunneling nanotubes or gap junctions that facilitate the exchange of substances between macrophages and β‐cells. The diabetic state upregulates the expression of PDGF in macrophages by AGEs, which activate the TLR4/MD2 receptor on macrophages and subsequently initiate transcription factors like NF‐κB. FFAs, free fatty acids; IL‐1β, interleukin‐1beta; TNF, tumor necrosis factor; NF‐κB, nuclear factor kappa B; JNK, c‐Jun N‐terminal kinase; ER, endoplasmic reticulum; GSIS, glucose‐stimulated insulin secretion; PDGF, platelet‐derived growth factor; AGEs, advanced glycation end‐products; TLR4, Toll‐like receptor 4; MD2, myeloid differentiation factor 2.

The functional impact of islet macrophages on β‐cells is mediated through multiple sophisticated communication mechanisms. Macrophages can sense β‐cell health by detecting ATP levels via purinergic receptors, triggering calcium responses and gene expression changes [5]. A critical mechanism involves direct cell–cell contact. Studies using coculture systems have shown that direct contact with macrophages from high‐fat‐diet‐fed mice significantly impairs glucose‐stimulated insulin secretion (GSIS), an effect not observed when contact is prevented [5]. Furthermore, exosomal transfer of microRNAs represents a key paracrine signaling pathway. Under metabolic stress, islet‐resident macrophages release exosomes containing miR‐155, which enters β‐cells and impairs insulin biosynthesis and secretion by targeting the PDX1 transcription factor [5]. Similarly, M1 macrophage‐derived exosomes can transfer miR‐212‐5p, which inhibits insulin secretion by regulating the Akt/GSK‐3β/β‐catenin pathway [8]. Emerging evidence also points to the role of TNTs. These transient membrane connections facilitate the direct transfer of cytoplasmic materials, including insulin secretory granules, from β‐cells to macrophages, a process that may be exacerbated in diabetic conditions. The interplay of these contact‐dependent and vesicle‐mediated mechanisms creates a complex regulatory network within the islet microenvironment that crucially influences β‐cell function and survival. The multimodal crosstalk between proinflammatory macrophages and β‐cells, including cytokine signaling, vesicle transfer, and direct cellular connections, is summarized in Figure 3.

#### 2.1.3 Metabolic Reprogramming, Epigenetic Regulation, and Therapeutic Targeting

Macrophage polarization is intrinsically linked to cellular metabolism, where a fundamental metabolic reprogramming underpins functional states: proinflammatory M1 subsets primarily rely on glycolysis, whereas anti‐inflammatory M2 subsets favor oxidative phosphorylation (OXPHOS) and fatty acid oxidation [9, 10]. This metabolic switch is critically influenced by the diabetic milieu, where factors such as hyperglycemia, advanced glycation end‐products (AGEs), and free fatty acids (FFAs) promote a shift toward the M1 phenotype via activation of pathways like NF‐κB and Toll‐like receptor (TLR)4, thereby creating a vicious cycle of inflammation and IR [11, 12]. Adding another layer of complexity, epigenetic regulation exerts precise control over macrophage polarization. Key enzymes, including histone deacetylases (HDACs) and demethylases such as Kdm5a, actively shape macrophage identity and function; for instance, HDAC inhibitors (e.g., butyrate) can restore histone acetylation and STAT1 signaling in diabetic macrophages [13], while Kdm5a activity has been associated with enhanced GSIS in islet macrophages. The crosstalk between carbon metabolism and epigenetic modifications (e.g., DNA methylation, histone modifications) further dynamically balances macrophage polarization [14].

The targeting of these regulatory nodes presents a promising therapeutic avenue. Existing medications, including metformin (which enhances OXPHOS and promotes M2 polarization) [12] and GLP‐1 receptor agonists, have demonstrated the capacity to promote an anti‐inflammatory M2 phenotype. Novel investigative approaches further expand this repertoire, revealing that dipeptidyl peptidase‐4 (DPP‐4) inhibitors like sitagliptin can shift macrophages from an M1 to an M2 state in diabetic bone repair [15], that specific probiotics can repair gut barrier function and modulate polarization via the TLR/MyD88/NF‐κB pathway, and that advanced biomaterials (e.g., immunomodulatory hydrogels) can spatially regulate macrophage phenotypes in diabetic wounds [16, 17]. CRISPR‐based gene editing and phytomedicine components targeting sirtuin‐mediated metabolic‐epigenetic axes also represent emerging strategies [14].

### 2.2 Mitochondrial Mechanisms in the Pathogenesis of T2D

Beyond immunometabolic dysregulation, mitochondrial dysfunction represents another core mechanism driving T2D progression. As metabolic hubs of β‐cells, mitochondria integrate nutrient signals into insulin secretion, with disrupted dynamics and impaired quality control directly contributing to β‐cell failure and systemic metabolic deterioration.

#### 2.2.1 Mitochondrial Dynamics and β‐Cell Dysfunction in T2D

Mitochondria are central to pancreatic β‐cell function, serving as metabolic hubs that convert nutrient signals into insulin secretion. In response to glucose, mitochondrial metabolism generates ATP, which triggers a cascade involving closure of ATP‐sensitive K^+^ channels, membrane depolarization, calcium influx, and insulin exocytosis [18]. β‐Cells are particularly vulnerable to oxidative stress due to high metabolic rates and low antioxidant defenses. In T2D, mitochondrial dynamics, the balance of fusion and fission events, are disrupted, leading to functional impairment. Fusion, mediated by proteins like mitofusins and optic atrophy 1, promotes mitochondrial content mixing, DNA stability, and repair. Fission, regulated by dynamin‐related protein 1 (DRP1) and mitochondrial fission factor, facilitates quality control and mitophagy [19]. In T2D, β‐cells exhibit fragmented mitochondria with reduced fusion, swollen morphology, and diminished membrane potential, as observed in human donors and diabetic models such as Goto‐Kakizaki rats [20]. This fragmentation impairs ATP production, amplifies reactive oxygen species (ROS) generation [21], and contributes to insulin secretory defects. Key regulators like AMP‐activated protein kinase (AMPK) influence dynamics by modulating peroxisome proliferator‐activated receptor gamma coactivator 1‐alpha (PGC1α), linking energy sensing to mitochondrial integrity [22]. Dysregulated dynamics also involve proteins such as SLC25A46, which promotes fission and mitigates lipotoxic stress, highlighting the role of mitochondrial morphology in β‐cell resilience. Thus, mitochondrial fragmentation emerges as a hallmark of T2D, driving oxidative stress and metabolic inefficiency.

#### 2.2.2 Mitophagy and Mitochondrial Quality Control in Diabetic β‐Cells

Mitophagy, the selective autophagy of damaged mitochondria, is critical for maintaining β‐cell health by eliminating dysfunctional organelles [23]. This process is governed by the PINK1–Parkin pathway, where PINK1 accumulation on impaired mitochondria recruits Parkin to ubiquitinate outer membrane proteins, targeting them for lysosomal degradation [24]. In T2D, chronic nutrient excess and glucolipotoxicity suppress mitophagy, leading to the accumulation of defective mitochondria, increased ROS, and apoptotic signaling [23]. Proteomic studies reveal altered expression of mitochondrial proteins in diabetes, underscoring defects in quality control [25]. Natural compounds like urolithin A, a metabolite of ellagic acid, have been shown to restore mitophagy and improve mitochondrial function in preclinical models [26]. Additionally, proteins such as DAP‐related apoptosis‐inducing kinase‐2 inhibit unc‐51‐like autophagy activating kinase 1, suppressing autophagy and exacerbating β‐cell dysfunction under high‐fat diet conditions [27]. Conversely, AMPK activation enhances mitophagy, promoting mitochondrial turnover and metabolic homeostasis [28].

The interplay between mitophagy and dynamics is evident: impaired fission disrupts the segregation of damaged mitochondria, while fusion defects hinder content repair [29]. Recent advances, including CRISPR‐based editing, highlight compartmentalized mitochondrial subpopulations in β‐cells such as hyperfused energy hubs versus fragmented ROS generators that adapt dynamically to metabolic fluctuations. Targeting mitophagy, through agents like metformin or novel compounds, represents a therapeutic strategy to preserve β‐cell mass and function in T2D.

#### 2.2.3 Interplay Between Macrophage Polarization and β‐Cell Mitochondrial Dynamics

In the context of T2D pathogenesis, the interplay between macrophage polarization and β‐cell mitochondrial dynamics represents a critical mechanism, where mitochondrial dynamics act as a molecular bridge in the crosstalk between immune cells and β‐cells [30]. Islet‐associated macrophages polarized toward a proinflammatory M1 phenotype release cytokines such as IL‐1β, which triggers mitochondrial fragmentation, excessive ROS production, and impaired GSIS in β‐cells [31]. In contrast, anti‐inflammatory M2 macrophages support mitochondrial fusion and facilitate tissue repair [32]. This bidirectional interaction is mediated through several mechanisms, including exosomal signaling, where M1 macrophage‐derived exosomes carry microRNAs like miR‐27‐3p that target mitochondrial dynamics proteins such as Miro1, leading to a state of fission dominance, impaired mitophagy, and NLRP3 inflammasome activation, with inhibition of miR‐27‐3p shown to prevent T2D progression in mouse models [33]. Additionally, metabolic reprogramming plays a role, as M1 macrophages depend on glycolysis and exhibit mitochondria prone to fission [33], while M2 phenotypes favor OXPHOS and mitochondrial fusion, with the AMPK and mTOR pathways modulating this metabolic–mitochondrial axis and influencing macrophage polarity [34]. Furthermore, organelle transfer via TNTs enables mitochondrial exchange between cells, such as stromal cells donating mitochondria to T cells, suggesting that similar intercellular exchanges may occur within pancreatic islets and influence β‐cell function [35].

### 2.3 Key Signaling Pathways in IR and β‐Cell Dysfunction

Beyond immunometabolic and mitochondrial mechanisms, the pathogenesis of T2D is driven by dysregulation in key signaling pathways. These intricate networks include insulin signaling, inflammatory cascades, ER stress, and nutrient‐sensing pathways, which integrate diverse metabolic and inflammatory signals to directly control insulin sensitivity and β‐cell function, offering critical targets for therapeutic intervention.

#### 2.3.1 Insulin Signaling Pathway and Its Dysregulation

Physiologically, insulin binding to its receptor activates the IRS/PI3K/Akt axis, a crucial cascade for maintaining metabolic homeostasis by promoting glucose uptake via the translocation of GLUT4, inhibiting gluconeogenesis through the phosphorylation and inactivation of the transcription factor FOXO1, and promoting glycogen synthesis by inactivating GSK [36]. Recent phosphoproteomic studies revealed that IR rewires this network through both attenuated phosphorylation at canonical sites (e.g., IRS‐1 Ser307) and emergence of unique phosphosites under insulin stimulation, suggesting dynamic network plasticity in metabolic tissues [36]. In T2D, however, chronic exposure to high glucose and nonesterified fatty acids (NEFAs) disrupts this pathway, as these metabolites and the ensuing inflammatory cytokines interfere with insulin receptor activation and subsequent IRS‐1/PI3K/Akt signaling, leading to IR. 2025 research identified PAQR3 as a key negative regulator of PI3K/AKT signaling, with therapeutic compounds like gentiopicroside shown to restore insulin sensitivity by disrupting PAQR3–PI3K interactions [37]. Additionally, HFD‐induced exosomes were found to suppress IRS‐2 expression, exacerbating pathway dysfunction [38], while novel crosstalk between NF‐κB/MAPK cascades and IRS‐1 phosphorylation was mechanistically linked to oxidative stress‐induced IR [39]. This disruption is a cornerstone of hyperglycemia, ultimately manifesting as increased glucose production by the liver and reduced glucose uptake in muscle and adipose tissue. Emerging evidence highlights tissue‐specific signaling rewiring, with adipocytes showing distinct phosphoproteomic signatures compared with skeletal muscle during IR progression [40].

#### 2.3.2 Inflammatory Signaling Pathways

Chronic, low‐grade inflammation represents a hallmark of obesity and T2D, primarily driven by macrophage infiltration into adipose tissue and the subsequent release of proinflammatory cytokines such as IL‐1β and TNF‐α, which collectively activate several interconnected signaling pathways that exacerbate IR. Recent studies identified a novel macrophage‐derived exosomal miRNA‐155 cluster that amplifies NF‐κB activation through direct targeting of IκBα ubiquitination, creating an additional layer of regulation in the inflammatory cascade [41, 42]. Key among these is the NF‐κB pathway, where excess metabolites and inflammatory factors activate the IKK complex, leading to NF‐κB's nuclear translocation; this not only amplifies the inflammatory response by upregulating cytokines like IL‐1β and TNF‐α in a positive feedback loop but also directly impairs insulin signaling by promoting the expression of inducible nitric oxide synthase, whose product, nitric oxide (NO), inhibits IRS‐1 activation, and by upregulating the NLRP3 inflammasome, which activates protein kinase C epsilon (PKCε) to further disrupt insulin signaling. A proteomics study revealed that PKCε‐mediated serine phosphorylation of IRS‐1 occurs preferentially at sites S307 and S636, with site‐specific phosphorylation patterns correlating with diabetes subtyping and cardiovascular comorbidity risk [43]. Simultaneously, the JAK/STAT pathway is engaged as cytokines like IL‐6 bind to their receptors, activating JAK and STAT proteins to upregulate the suppressor of cytokine signaling 3 (SOCS3), which directly inhibits the IRS‐1 cascade and exhibits crosstalk that promotes the NF‐κB pathway. New evidence demonstrates that SOCS3 forms a ternary complex with TLR4 and MyD88 in adipose tissue macrophages, mechanistically linking innate immune signaling to IR [44, 45]. Furthermore, inflammatory factors and metabolic stress activate c‐Jun N‐terminal kinase (JNK), which phosphorylates IRS‐1 on serine residues to block its proper activation and concurrently promotes gluconeogenesis by phosphorylating and driving the nuclear translocation of FOXO1. Cutting‐edge single‐cell RNA sequencing has uncovered a distinct subpopulation of JNK‐hyperactivated adipocytes that secrete extracellular vesicles (EVs) containing miR‐27a‐3p, which propagates IR to hepatocytes via the FOXO1/PGC‐1α axis [46]. These inflammatory pathways are not only central to the development of IR but also contribute significantly to the progression of metabolic dysfunction syndrome (MDS)‐related target organ damage, including diabetic nephropathy, retinopathy, and impaired wound healing in diabetic foot ulcers (DFUs). A clinical trial targeting the NLRP3–IL‐1β axis with combination therapy (canakinumab + metformin) showed 32% greater improvement in insulin sensitivity compared with metformin alone, particularly in the severe insulin‐resistant diabetes subtype [47, 48].

#### 2.3.3 Endoplasmic Reticulum Stress

In T2D, the high metabolic demand on pancreatic β‐cells and chronic nutrient excess in insulin‐sensitive tissues overwhelm the protein‐folding capacity of the endoplasmic reticulum (ER), leading to the accumulation of misfolded proteins and triggering a state known as ER stress (ERS). This cellular crisis activates the unfolded protein response (UPR) through its key sensors: PERK, IRE1α, and ATF6. The PERK pathway, by phosphorylating eIF2α, increases the translation of the transcription factor ATF4, which subsequently upregulates the proapoptotic factor CHOP and Tribbles homolog 3 (TRIB3); TRIB3 directly inhibits Akt activity, thereby promoting IR [49, 50]. Simultaneously, the IRE1α pathway, upon binding with TRAF2, activates IKK and JNK1, creating a critical link that integrates ERS with inflammatory signaling and further exacerbates IR [51, 52]. Recent studies highlight that ER stress can propagate intercellularly via connexin in hepatocytes, amplifying hepatic IR and steatosis [51]. Additionally, mitochondrial‐associated membranes dysfunction has been causally linked to hepatic ER stress‐induced IR in obesity models [53, 54]. When ERS is sustained, it ultimately initiates apoptotic pathways in β‐cells through transcriptional reprogramming and loss of cellular identity [55], contributing directly to their progressive loss. This mechanism is also implicated in diabetic complications, where ER stress‐induced β‐cell dedifferentiation precedes functional deficits [56]. Notably, pharmacological interventions targeting ER homeostasis (e.g., 4‐PBA) or enhancing chaperone activity show potential in reversing IR [57, 58], though therapeutic applications remain to be investigated [59].

#### 2.3.4 Oxidative Stress

An imbalance between ROS production and antioxidant defenses characterizes oxidative stress. Mitochondrial dysfunction in skeletal muscle and other tissues leads to increased ROS, which exacerbates IR through the activation of JNK and NF‐κB pathways [60]. Emerging human studies using invasive in vivo approaches confirm mitochondrial redox state manipulation directly affects muscle insulin action, validating preclinical models [60]. In pancreatic β‐cells, which have low antioxidant defense, oxidative stress causes mitochondrial and DNA damage, impairing ATP production and insulin secretion [61, 62]. Recent findings highlight Nrf2's critical role in counteracting β‐cell oxidative stress through regulating survival pathways [62], while selenium‐based therapies show promise in restoring endogenous antioxidant capacity [63]. It also downregulates key transcription factors like PDX1 and MAFA, reducing insulin synthesis, with new evidence showing AMPK activation can mitigate this effect via NRF2 nuclear translocation [64]. ALDH2 activation has also been shown to improve β‐cell function through HS–AMPK–G6PD signaling under high‐glucose conditions [65].

#### 2.3.5 Nutrient‐Sensing and Metabolic Pathways

In the context of T2D, nutrient‐sensing and metabolic pathways play pivotal yet opposing roles in the regulation of insulin sensitivity and metabolic homeostasis. The mTOR pathway, chronically activated by nutrient excess, paradoxically contributes to IR by phosphorylating IRS‐1 on serine residues, thereby blocking its function, while simultaneously promoting hepatic lipogenesis through the activation of sterol regulatory element‐binding protein (SREBP) [66]. Recent studies highlight that mTORC1‐mediated suppression of autophagy exacerbates pancreatic β‐cell dysfunction under glucolipotoxic conditions, further impairing insulin secretion [67]. Similarly, increased Notch signaling exacerbates metabolic dysfunction by inducing the expression of SREBP1 to promote hepatic fat deposition and upregulating gluconeogenic enzymes in a FOXO1‐dependent manner [68], with new evidence showing intestinal Notch activation impairs GLP‐1 secretion, worsening postprandial glucose control [69].

In stark contrast, the AMPK pathway, a central regulator of energy homeostasis that normally promotes glucose uptake and suppresses gluconeogenesis, is significantly suppressed in T2D due to decreased adiponectin levels and chronic inflammation [70]. This suppression of AMPK activity not only contributes to hyperglycemia and hyperlipidemia but also impairs critical quality control mechanisms such as mitophagy, thereby weakening the clearance of damaged cellular components. Emerging work demonstrates that AMPK activation via time‐restricted eating (TRE) improves hepatic glycogen metabolism and insulin sensitivity in T2D patients, though its long‐term efficacy remains under investigation [71].

The dysregulated mTOR activation demonstrates context‐dependent effects by reducing antioxidant activity in the kidneys yet promoting wound healing in DFUs [72]. Notably, recent findings reveal that RhoGDIα overexpression in skeletal muscle exacerbates IR by inhibiting Rac1 activity, while its knockdown enhances whole‐body glucose homeostasis [73]. Additionally, exosomal miRNAs from M2 macrophages have been shown to improve insulin sensitivity in obese mice, offering a novel therapeutic avenue [74].

#### 2.3.6 Signaling Pathways With Context‐Dependent or Dual Roles in T2D

Several signaling pathways exhibit complex and often context‐dependent roles in the pathogenesis of T2D. The Wnt pathway demonstrates divergent effects, where the ligand Wnt5a can aggravate IR by activating noncanonical pathways that engage JNK signaling, whereas Wnt3a may conversely improve insulin sensitivity and inhibit adipocyte differentiation. Recent studies have identified RSPO1 as a key agonist of the Wnt/β‐catenin pathway that promotes β‐cell neogenesis and replication, counteracting chemically induced or autoimmune‐mediated diabetes [75]. Additionally, Wnt4 has been shown to regulate calcium signaling and metabolic pathways in β‐cells during postnatal maturation [76], while hyperactive Wnt/β‐catenin signaling in diabetic kidney disease contributes to renal fibrosis and can be disrupted by the peptide KP6 through binding to Wnt ligands [77]. This highlights the Wnt pathway's dual nature, where its regenerative benefits in β‐cells are counterbalanced by its fibrotic effects in the kidney, complicating its therapeutic targeting.

Similarly, the hypoxia‐inducible factor (HIF) pathway functions as a double‐edged sword; while it may potentially improve IR in adipose tissue by stimulating thermogenic activity, it simultaneously transcriptionally activates SOCS3, which inhibits insulin signaling. In diabetic conditions, HIF‐1α protein is present in pancreatic β‐cells and its inhibition with PX‐478 improves β‐cell function by enhancing insulin secretion and reducing dedifferentiation markers [78]. HIF‐1α also interacts with β‐catenin to promote glycolysis‐dependent inflammation in alveolar macrophages while suppressing mitochondrial metabolism [79]. In diabetic retinopathy, YAP/TAZ are O‐GlcNAcylated at threonine 383, leading to stabilization and activation that promotes vascular dysfunction through proangiogenic and glucose metabolic programs [80]. However, the same mTORC1 signaling is physiologically indispensable for compensatory β‐cell proliferation and survival in response to IR [81]. This creates a significant therapeutic challenge: systemic mTOR inhibition could improve insulin sensitivity but might simultaneously impair the very β‐cell compensation needed to maintain glucose homeostasis.

Furthermore, the Hippo/YAP pathway, a key regulator of cell proliferation and differentiation, promotes glucose uptake, glycolysis, and lipogenesis. Under diabetic conditions, LATS2 (a core Hippo component) is activated and induces β‐cell apoptosis [82], while YAP inactivation exacerbates diabetic kidney injury by reducing WT1 expression in podocytes [83]. In mesangial cells, high glucose directly activates YAP/TAZ through the canonical Hippo pathway, recapitulating diabetic nephropathy hallmarks like extracellular matrix deposition [84]. The pathway also shows crosstalk with HIF‐1α, where hypoxia induces HIF‐1α‐mediated YAP upregulation by reducing YAP phosphorylation and promoting its nuclear localization [85, 86]. This HIF‐1α‐YAP interaction enhances DNA damage repair in glioblastoma cells [87], highlighting the intricate and sometimes paradoxical nature of these regulatory networks in glucose homeostasis.

#### 2.3.7 Central IR and Neuroendocrine Dysregulation

Central IR represents a pivotal yet often overlooked dimension in the pathogenesis of T2D, linking peripheral metabolic disturbances with neuroendocrine and behavioral dysregulation [88]. In the brain, particularly within the hypothalamus, impaired insulin signaling disrupts the normal regulation of energy homeostasis, leading to appetite dysregulation [88]. This manifests as reduced satiety signaling and heightened preference for calorie‐dense foods, which perpetuates caloric excess and weight gain, key drivers of peripheral IR and β‐cell stress [89]. Concurrently, dysregulated insulin action in the central nervous system (CNS) contributes to altered sympathetic nervous system (SNS) outflow [88]. Elevated SNS activity not only promotes hypertension and cardiovascular complications but also exacerbates hepatic glucose production and lipolysis, further fueling hyperglycemia and dyslipidemia [90]. This neuroendocrine imbalance creates a feed‐forward cycle: peripheral IR and inflammation impair central insulin action, which in turn worsens systemic metabolic control via behavioral and autonomic pathways [88]. Emerging evidence also suggests that SGLT2 inhibitors (e.g., empagliflozin, dapagliflozin) and GLP‐1 receptor agonists may partly exert their benefits by modulating central insulin sensitivity and SNS tone [91], highlighting the brain as a therapeutic target in T2D [88]. Thus, central IR, appetite dysregulation, and sympathetic overactivity form an integral triad that amplifies and sustains the systemic metabolic dysfunction characteristic of T2D.

#### 2.3.8 Major Knowledge Gaps, Methodological Challenges, and Unresolved Controversies

Despite remarkable progress, several controversies and critical knowledge gaps persist in our understanding of T2D pathogenesis. First, the role of nutrient sensing and developmental pathways is highly dependent on the environment and may even be contradictory. As described in Section 2.3.6, although chronic mTORC1 overactivation leads to IR through IRS‐1 serine phosphorylation, its physiological signal is also necessary to compensate for β‐cell proliferation and survival, which poses a significant therapeutic challenge [67, 81]. In a similar vein, the Wnt pathway exhibits divergent functions: the ligand Wnt3a may improve insulin sensitivity, whereas Wnt5a aggravates IR by activating JNK; moreover, while hyperactive Wnt/β‐catenin signaling worsens diabetic kidney disease, it is also indispensable for β‐cell neogenesis [75, 77]. The HIF pathway is another double‐edged sword, as HIF‐1α inhibition with PX‐478 can preserve β‐cell function, yet HIF‐1α‐driven glycolysis is necessary to sustain macrophage inflammatory responses, complicating straightforward therapeutic targeting [78, 79]. Second, there is still a major translation gap in transferring research results from rodent models to human pathophysiology, particularly in terms of mitochondrial dynamics and immune metabolism crosstalk. The seminal research on mitochondrial fission, fusion, and mitophagy largely relies on genetic mouse models, which may not reproduce the chronic, lifestyle driven properties of human T2D [92]. Likewise, the emerging modalities of exosome‐mediated and nanotube‐dependent macrophage–β‐cell communication have been characterized almost exclusively in vitro or in mice, with only sparse validation in primary human islets [93]. These preclinical–clinical discrepancies are reflected in the mixed or disappointing outcomes of several anti‐inflammatory trials, underscoring the difficulty of translating pathway‐centric findings into effective human therapies. Third, the relative contribution of β‐cell dedifferentiation versus apoptosis to the progressive loss of functional β‐cell mass in human T2D remains intensely debated. Single‐cell and multiomics analyses have revealed a spectrum of dysfunctional β‐cell states marked by downregulation of PDX1 and MAFA, suggesting that dedifferentiation is an early and potentially reversible event; however, accurately quantifying its contribution relative to apoptotic cell death in vivo remains technically challenging [56, 94]. Fourth, under the condition of diabetes, the hierarchical relationship and crosstalk between key stress signal cascades (including inflammation, ERS, and oxidative stress) in human islets are still unclear. Although animal models indicate that they are globally activated, there is still a lack of comprehensive research on simultaneously mapping these pathways in human samples at different disease stages [52]. Addressing these interconnected controversies through advanced human‐relevant models and multiomics integration represents a critical frontier that will be essential for developing therapies that target upstream master regulatory nodes.

### 2.4 Pancreatic β‐Cell Dysfunction: A Critical Determinant

While IR in peripheral tissues establishes the foundational metabolic milieu for T2D, it is the subsequent failure of the pancreatic β‐cell to compensate that ultimately dictates the progression from normoglycemia to clinical hyperglycemia. Initially, β‐cells mount a robust compensatory response to IR, characterized by hyperinsulinemia that maintains glucose homeostasis. However, this compensatory state is not sustainable.

#### 2.4.1 Dedifferentiation and Transdifferentiation

A paradigm shift in understanding β‐cell failure in T2D has been the recognition that the loss of functional β‐cell mass is not solely due to apoptosis but also involves the processes of dedifferentiation and transdifferentiation. Under persistent metabolic stress from glucotoxicity and lipotoxicity, mature β‐cells undergo a dramatic loss of their specific identity. Key transcription factors that define and maintain β‐cell maturity and function, such as PDX1, MAFA, and NKX6.1, are significantly downregulated [94, 95]. This erosion of the transcriptional landscape causes β‐cells to revert to a progenitor‐like, nonfunctional state, ceasing insulin production. Recent studies identified SMOC1 as a novel inducer of β‐cell dysfunction and dedifferentiation [96], while BRD4 was found to modulate β‐cell differentiation states in diabetic models [97]. In more extreme cases, some β‐cells transdifferentiate into other endocrine cell types, most notably glucagon‐producing α‐cells or somatostatin‐producing δ‐cells [98]. Single‐cell RNA‐seq studies revealed disharmonic remodeling of β‐cells in T2D, challenging linear transdifferentiation trajectories, while SARS‐CoV‐2 infection was shown to induce eIF2‐pathway‐mediated β‐cell transdifferentiation [99]. This fate switch further exacerbates hyperglycemia by not only reducing insulin output but also potentially increasing the relative secretion of counter‐regulatory hormones. The driver of this process includes the activation of stress‐response pathways and the re‐expression of embryonic transcription factors like ALDH1A3 [95], which is normally silenced in mature islets. METRNL protein has been implicated in modulating this process, and Txnip inhibition was shown to prevent dedifferentiation in β‐cell failure models [56]. This mechanism of functional mass loss, where β‐cells are present but no longer operational, presents a potential therapeutic opportunity, as these cells might be coaxed back to a mature, insulin‐producing state, unlike those lost to apoptosis.

#### 2.4.2 Cellular Senescence and Nonapoptotic Cell Death

Beyond traditional apoptosis, β‐cells in T2D are susceptible to cellular senescence and alternative forms of programmed cell death, which perpetuate a local inflammatory milieu and accelerate dysfunction. Cellular senescence is an irreversible state of cell cycle arrest triggered by chronic hyperglycemia, oxidative stress, and DNA damage. Recent studies reveal that senescent human β‐cells exhibit chromatin reorganization activating enhancers for functional maturation genes, paradoxically showing elevated GSIS capacity despite their senescent state [100]. While this paradoxical enhancement of GSIS has been observed in isolated β‐cells, it is accompanied by a deleterious senescence‐associated secretory phenotype (SASP) that propagates inflammation and impairs the function of neighboring β‐cells, ultimately compromising overall islet function and accelerating β‐cell failure. Notably, interferon‐stimulated genes are upregulated in senescent human β‐cells, whereas classic SASP cytokine genes remain unchanged, suggesting a unique inflammatory signature [100]. This SASP creates a toxic local environment that impairs the function of neighboring healthy β‐cells and promotes immune cell infiltration, establishing a vicious cycle of inflammation and damage.

Furthermore, nonapoptotic cell death pathways are prominently activated. Pyroptosis, an intensely inflammatory form of cell death, is initiated by the NLRP3 inflammasome in response to metabolic stressors. Emerging evidence shows pyroptosis‐driven EVs and IL‐1β secretion can induce senescence in neighboring stem cells [101], suggesting bidirectional crosstalk between pyroptosis and senescence pathways. Activation of NLRP3 leads to the cleavage of caspase‐1, which gasdermin D to form pores in the plasma membrane, resulting in cell lysis and the release of mature IL‐1β and IL‐18, thus amplifying islet inflammation. Recent work demonstrates that chemotherapy‐induced senescence‐to‐pyroptosis transition promotes disease recurrence in other cell types [102], highlighting the dynamic interplay between these processes. Separately, ferroptosis, an iron‐dependent process driven by the accumulation of lipid peroxides, has been implicated in β‐cell loss. The unique susceptibility of β‐cells to oxidative stress and their relatively low expression of antioxidant defense genes, particularly those guarding against lipid peroxidation like GPX4, makes them vulnerable to this form of destruction [103]. New therapeutic strategies targeting mPGES‐2 via the PGE–EP3–NR4A1 axis have shown promise in reducing β‐cell senescence and dysfunction [104], while exercise‐induced AMPK activation was found to decrease β‐cell senescence markers through NRF2 nuclear translocation [64].

#### 2.4.3 Impairment of Insulin Secretory Mechanisms

The core functional defect in T2D is the failure of β‐cells to secrete insulin appropriately in response to glucose, a process governed by exquisitely regulated signaling pathways that are directly impaired by the diabetic environment. Recent studies reveal that β‐cell senescence exacerbates this dysfunction, with p16‐positive β‐cells showing impaired insulin secretion capacity and elevated interferon responses, while paradoxically lacking typical SASP cytokine expression [100]. The canonical KATP channel‐dependent pathway begins with glucose uptake, leading to an increased ATP/ADP ratio. This closes ATP‐sensitive K^+^ (KATP) channels, causing membrane depolarization, opening of voltage‐gated Ca^2+^ channels, and a surge in cytosolic Ca^2+^ that triggers insulin granule exocytosis. In T2D, chronic hyperglycemia can lead to mitochondrial dysfunction, reducing ATP production and blunting this pathway. Notably, ferroptosis has emerged as a key regulator of cellular senescence during aging, with its inhibition shown to delay aging and improve healthspan, potentially impacting β‐cell function [105]. More nuanced is the failure of KATP channel‐independent amplification pathways, which are crucial for the full secretory response. These pathways, potentiated by incretin hormones like GLP‐1, involve metabolic coupling factors that sensitize the secretory machinery to Ca^2+^. Key among these regulators is AMPK, whose activity is dysregulated in T2D, disrupting the energy‐sensing apparatus that coordinates insulin release. Exercise‐induced AMPK activation has been shown to reduce β‐cell senescence markers through NRF2 nuclear translocation [64]. Furthermore, glucolipotoxicity disrupts mitochondrial function, not only limiting ATP but also generating excessive ROS that damage mitochondrial DNA and proteins. This compromises the finely tuned oscillations in Ca^2+^ signaling and deprives the exocytosis process of its essential metabolic signals, leading to a specific defect in GSIS while often leaving the basal secretion intact or even elevated. The crosstalk between ferroptosis and pyroptosis via autophagy may also contribute to β‐cell dysfunction under diabetic conditions, though the exact mechanisms remain unclear [106, 107].

**FIGURE 4:**
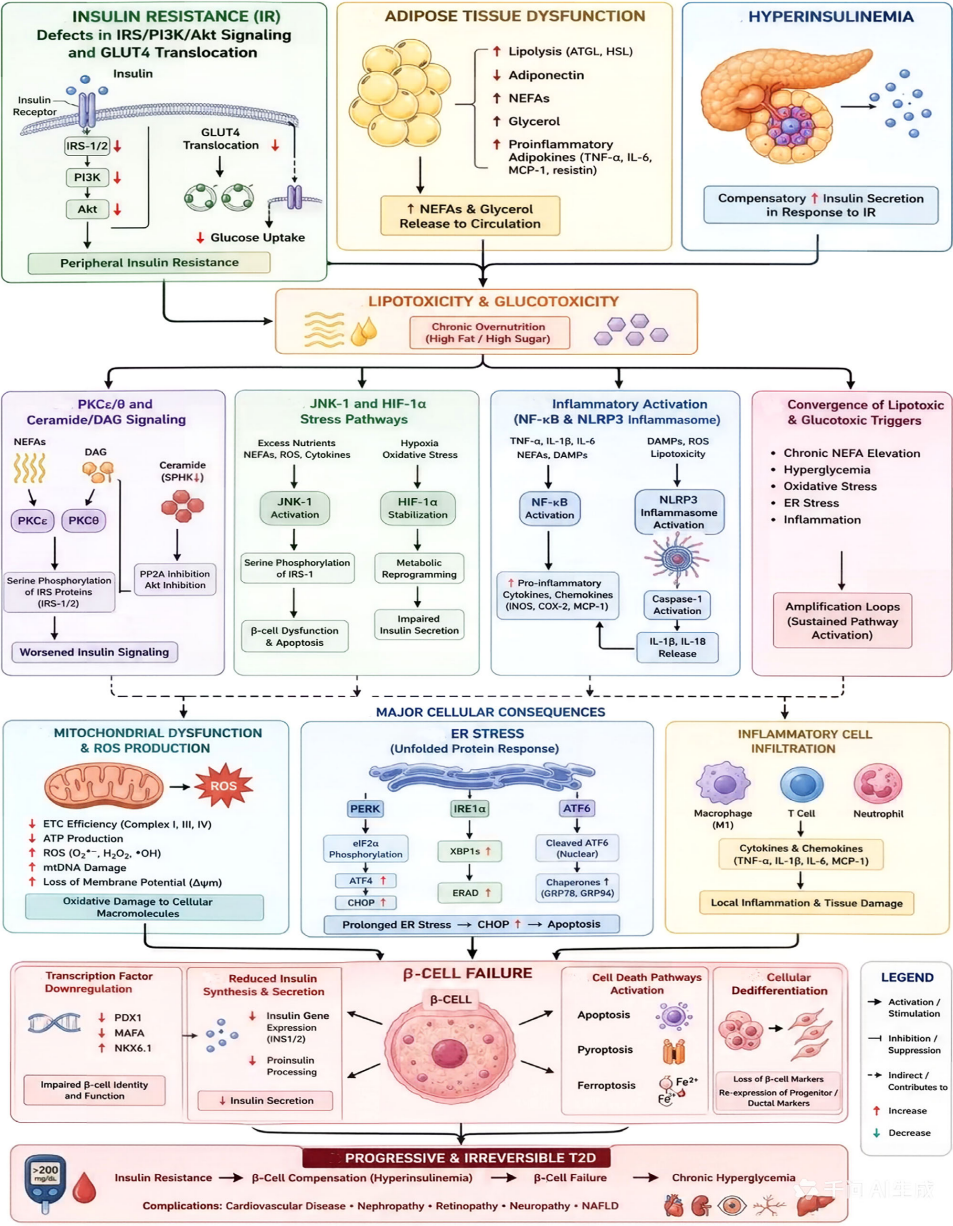
Progressive mechanisms of insulin resistance, lipotoxicity, inflammation, and β‐Cell failure in T2D. This schematic illustrates the key molecular mechanisms driving T2D progression, beginning with IR characterized by defects in IRS/PI3K/Akt signaling and GLUT4 translocation, alongside adipose tissue dysfunction marked by increased lipolysis, reduced adiponectin, and elevated release of nonesterified fatty acids (NEFAs) and glycerol. These disturbances lead to hyperinsulinemia and establish a state of lipotoxicity and glucotoxicity, which in turn activate central pathogenic pathways including PKCε/θ and ceramide/DAG signaling, JNK‐1 and HIF‐1α stress pathways, and inflammatory activation of NF‐κB and the NLRP3 inflammasome. These pathways collectively induce mitochondrial dysfunction and ROS production, ER stress via PERK, IRE1α, and ATF6 sensors, and inflammatory cell infiltration. The convergence of these effects ultimately causes β‐cell failure through downregulation of key transcription factors such as PDX1 and MAFA, reduced insulin synthesis, activation of apoptosis, pyroptosis, and ferroptosis, as well as cellular dedifferentiation, resulting in progressive and irreversible T2D. IRS, insulin receptor substrate; PI3K, phosphoinositide 3‐kinase; Akt, protein kinase B; GLUT4, glucose transporter Type 4; NEFAs, nonesterified fatty acids; PKCε/θ, protein kinase C epsilon/theta; DAG, diacylglycerol; JNK‐1, c‐Jun N‐terminal kinase‐1; HIF‐1α, hypoxia‐inducible factor 1‐alpha; NF‐κB, nuclear factor kappa B; NLRP3, NLR family pyrin domain containing 3; ROS, reactive oxygen species; ER, endoplasmic reticulum; PERK, PKR‐like ER kinase; IRE1α, inositol‐requiring enzyme 1 alpha; ATF6, activating transcription factor 6; PDX1, pancreatic and duodenal homeobox 1; MAFA, MAF BZIP transcription factor A.

In summary, the molecular pathogenesis of T2D is driven by the synergistic interplay of immunometabolic dysregulation, mitochondrial dysfunction, and lipotoxic signaling. These core mechanisms collectively impair insulin signal transduction and erode pancreatic β‐cell functional mass, thereby establishing the fundamental molecular basis for the transition from IR to overt hyperglycemia. A summary of these key mechanisms is provided in Table 1. The sequential progression and causal relationships between these pathways are integrated into Figure 4, which illustrates the progressive mechanisms of IR, lipotoxicity, inflammation, and β‐cell failure in T2D.

**TABLE 1:** Key molecular mechanisms in the pathogenesis of T2D.

Pathogenic mechanism	Key molecules/processes	Role in T2D pathogenesis	References
Immunometabolic axis	Macrophage polarization (M1/M2 spectrum)	Drives inflammation, β‐cell dysfunction, and insulin resistance via cytokine release and exosomal miRNA transfer	[3, 4, 5, 6, 7]
	Exosomal miRNAs (miR‐155, miR‐212‐5p)	Impair insulin biosynthesis and secretion by targeting PDX1 and Akt/GSK‐3β/β‐catenin pathways	[5, 8]
	Tunneling nanotubes (TNTs)	Facilitate intercellular transfer of insulin granules and mitochondrial material, exacerbating β‐cell stress	[5, 8]
Mitochondrial dysfunction	Altered dynamics (fusion/fission imbalance)	Leads to fragmented mitochondria, reduced ATP production, and increased ROS, impairing insulin secretion	[20, 21, 22, 23]
	Impaired mitophagy (PINK1–Parkin pathway)	Accumulation of damaged mitochondria, promoting oxidative stress and apoptosis in β‐cells	[24, 25, 26, 27]
Lipotoxicity	Ceramides	Inhibit Akt signaling via PP2A/PKCζ, induce mitochondrial apoptosis, and contribute to IR and β‐cell loss	[108, 109, 110, 111, 112, 113]
	Diacylglycerols (DAGs)	Activate novel PKC isoforms (ε, θ), leading to inhibitory serine phosphorylation of IRS and impaired insulin signaling	[114, 115, 116]
	Disrupted cholesterol homeostasis	Alters membrane lipid raft integrity, impairing insulin receptor signaling and insulin granule exocytosis	[117, 118, 119, 120]
Proteotoxicity	Islet amyloid polypeptide (IAPP) aggregation	Forms toxic oligomers that disrupt membrane integrity, induce ER stress, and activate NLRP3 inflammasome	[139, 140, 141, 142, 143]
	Impaired autophagy	Failure to clear damaged organelles and protein aggregates, accelerating β‐cell dysfunction and apoptosis	[81, 144, 145]
Inflammatory signaling	NF‐κB and JNK pathways	Promote serine phosphorylation of IRS, upregulate SOCS3, and enhance proinflammatory cytokine production	[41, 42, 43, 44, 45, 46]
	NLRP3 inflammasome	Activated by metabolic stressors, leading to caspase‐1‐mediated pyroptosis and IL‐1β release in islets	[47, 48]
ER stress and UPR	PERK, IRE1α, ATF6 pathways	Trigger apoptosis and inhibit insulin signaling via CHOP, TRIB3, and JNK/IKK activation under nutrient overload	[49, 50, 51, 52, 53, 54, 55, 56, 57, 58, 59]
Oxidative stress	ROS overproduction	Damages mitochondrial DNA and proteins, impairs insulin secretion, and promotes β‐cell senescence	[60, 61, 62, 63, 64, 65]
Nutrient‐sensing pathways	mTOR overactivation	Promotes IRS‐1 serine phosphorylation, suppresses autophagy, and exacerbates lipogenesis and IR	[66, 67]
	AMPK suppression	Reduces glucose uptake, impairs mitophagy, and contributes to hyperglycemia and lipid accumulation	[70, 71]
Gut–metabolism axis	Short‐chain fatty acids (SCFAs)	Microbial metabolites that enhance insulin sensitivity, stimulate GLP‐1 secretion, and maintain gut barrier integrity	[146, 147, 148, 149, 150]
	Branched‐chain amino acids (BCAAs)	Elevated levels impair mitochondrial function and activate mTORC1, contributing to insulin resistance	[151, 152, 153, 154, 155]
	Secondary bile acids	Activate FXR and TGR5 receptors to modulate glucose homeostasis, GLP‐1 secretion, and hepatic lipogenesis	[156, 157, 158, 159, 160, 161]
β‐Cell dysfunction	Dedifferentiation (loss of PDX1, MAFA, NKX6.1)	Reversion to progenitor‐like state, ceasing insulin production and promoting transdifferentiation	[56, 94, 95, 96, 97, 98, 99]
	Cellular senescence and SASP	Irreversible cell cycle arrest with proinflammatory secretory phenotype, impairing neighboring β‐cell function	[100, 101, 102, 103, 104, 105]
	Pyroptosis and ferroptosis	Inflammatory and iron‐dependent cell death pathways activated by metabolic stress and oxidative damage	[101, 102, 103, 104, 105, 106, 107]

Abbreviations: Akt, protein kinase B; AMPK, AMP‐activated protein kinase; ATF6, activating transcription factor 6; ATP, adenosine triphosphate; BCAAs, branched‐chain amino acids; CHOP, C/EBP homologous protein; DAGs, diacylglycerols; ER, endoplasmic reticulum; FXR, farnesoid X receptor; GLP‐1, glucagon‐like peptide‐1; GSK‐3β, glycogen synthase kinase‐3 beta; IAPP, islet amyloid polypeptide; IKK, IκB kinase; IRE1α, inositol‐requiring enzyme 1 alpha; IRS, insulin receptor substrate; JNK, c‐Jun N‐terminal kinase; M1, classically activated macrophages; M2, alternatively activated macrophages; MAFA, MAF BZIP transcription factor A; miR‐155, microRNA‐155; miR‐212‐5p, microRNA‐212‐5p; miRNAs, microRNAs; mTOR, mechanistic target of rapamycin; NF‐κB, nuclear factor kappa B; NKX6.1, NK6 homeobox 1; NLRP3, NLR family pyrin domain containing 3; PDX1, pancreatic and duodenal homeobox 1; PERK, PKR‐like ER kinase; PINK1, PTEN‐induced putative kinase 1; PKC, protein kinase C; PKCζ, protein kinase C zeta; PP2A, protein phosphatase 2A; ROS, reactive oxygen species; SASP, senescence‐associated secretory phenotype; SCFAs, short‐chain fatty acids; SOCS3, suppressor of cytokine signaling 3; TGR5, Takeda G protein‐coupled receptor 5; TNTs, tunneling nanotubes; TRIB3, tribbles homolog 3; UPR, unfolded protein response.

## 3 Systemic Metabolic Derangements and Tissue‐Specific Pathologies

While the fundamental molecular insults: from dysregulated immune cells and faltering organelles to stress signaling pathways within the β‐cell where these insults do not occur in isolation. They collectively drive a systemic metabolic breakdown, where the specific molecular players, such as ceramides and DAGs, find their pathogenic expression in a tissue‐specific manner. The liver, skeletal muscle, and pancreas become stages for these processes, where ectopic lipid deposition, proteostatic collapse, and a perturbed gut–metabolism axis converge to create the distinctive and self‐perpetuating pathophysiology of T2D.

### 3.1 Ectopic Lipid Deposition and Lipotoxicity

While inflammatory and nutrient–stress pathways provide a fundamental framework for understanding T2D pathogenesis, the specific molecular mediators translating lipid oversupply into cellular dysfunction warrant focused examination. The concept of “lipotoxicity” extends beyond mere fat accumulation, describing the pathogenic effects of specific lipid species that ectopically deposit in nonadipose tissues. This ectopic lipid deposition is not a passive process but an active driver of disease, where distinct lipid molecules like ceramides, DAG, and disrupted cholesterol directly impair insulin signaling and β‐cell function through discrete yet interconnected mechanisms, cementing the vicious cycle of metabolic deterioration.

#### 3.1.1 Ceramides: The Core Apoptotic and Insulin‐Antagonistic Lipid

Among the most potent lipotoxic molecules are ceramides, sphingolipids that act as central mediators of both IR and β‐cell apoptosis. Their accumulation is a critical step in lipid‐induced metabolic dysfunction, with recent studies demonstrating that limiting intracellular ceramide accumulation in human pluripotent stem cell‐derived β cells (hPSC‐β cells) remarkably enhances insulin processing and GSIS [108]. Ceramides exert their deleterious effects through multiple, parallel mechanisms. Primarily, they directly inhibit the pivotal Akt/PKB signaling pathway. This is achieved by activating protein phosphatase 2A (PP2A), which dephosphorylates and inactivates Akt, and by promoting the activity of atypical protein kinase C ζ (PKCζ), which further disrupts insulin signal transduction [109]. Recent work has shown that SIRT1 can restore insulin secretory function in β‐cells by reducing proapoptotic ceramide synthesis through deacetylation of TLR4, thereby decreasing sphingolipid synthesis enzymes SPTLC1/2 via AKT/NF‐κB pathway [110]. Consequently, key metabolic processes like GLUT4 translocation and glycogen synthesis are halted.

Beyond insulin signaling, ceramides are powerful inducers of mitochondrial‐mediated apoptosis. They facilitate the formation of permeable pores in the mitochondrial outer membrane, promoting the release of cytochrome *c* and activation of executioner caspases. New evidence reveals that inflammatory mitochondria from macrophages can fuse with β‐cell mitochondria, causing lipid peroxidation and mtDNA release that activates STING pathway‐mediated apoptosis [111].

Additionally, ceramides derived from astrocytes via de novo synthesis pathway (regulated by SPTLC2) have been shown to contribute significantly to cellular apoptosis [112]. This dual role, impairing insulin action while simultaneously triggering cell death, establishes ceramides as a core pathological link between lipid oversupply, peripheral IR, and the loss of functional β‐cell mass in T2D. Recent clinical studies have further demonstrated that circulating ceramide levels can predict diabetes remission after bariatric surgery independent of weight loss [113], highlighting their potential as both biomarkers and therapeutic targets in metabolic disorders.

#### 3.1.2 Activator of Protein Kinase C and Insulin Receptor Substrate Serine Phosphorylation

The accumulation of DAGs in nonadipose tissues like liver, skeletal muscle, and pancreatic islets is another major driver of lipotoxicity. DAG functions as a potent second messenger that activates several isoforms of the PKC family, with structural studies revealing how C1 domains in PKC isoforms recognize and capture DAGs in biological membranes [114]. In the context of IR, the novel PKC isoforms ε and δ are particularly relevant, as demonstrated by their involvement in hepatic steatosis and IR through the plasma membrane sn‐1,2‐DAGs–PKCε–IRK pathway [115]. In the liver and muscle, DAG‐mediated activation of PKCε leads to the serine phosphorylation of the insulin receptor substrate (IRS) proteins, with evidence showing this pathway is modulated by mitochondrial fat oxidation [115]. This phosphorylation event blocks insulin signal transduction. Within pancreatic β‐cells, the activation of PKC by DAG contributes to metabolic dysfunction, as shown by studies detecting endogenous PKC activity in cellular organelles [116]. Thus, DAG serves as a key lipid intermediate that disrupts insulin sensitivity in metabolic tissues, primarily through PKC signaling cascades.

#### 3.1.3 Cholesterol Homeostasis Disruption: Impacts on Membrane Integrity and Secretory Function

Dysregulated cellular cholesterol metabolism is an increasingly recognized facet of lipotoxicity that impairs both insulin action and secretion. In insulin‐sensitive cells, cholesterol is a critical component of lipid rafts, specialized membrane microdomains where the insulin receptor signaling complex is assembled. Excessive cholesterol accumulation disrupts the ordered structure of these rafts, impairing insulin receptor dimerization and its subsequent signaling efficacy [117]. In pancreatic β‐cells, cholesterol homeostasis is paramount for the insulin secretory process. Elevated intracellular cholesterol directly interferes with the fusion and exocytosis of insulin granules [118]. Furthermore, it disrupts the function of glucose transporters and ion channels embedded in the plasma membrane. The regulation of cholesterol influx, synthesis via the SCAP–SREBP2 pathway, and efflux through transporters like ABCA1 is crucial [119]. Polymorphisms in genes governing these processes are linked to an increased risk of T2D [120], highlighting the importance of maintaining precise cholesterol balance for proper β‐cell function and systemic glucose homeostasis.

### 3.2 Organ‐Specific Effects of Ectopic Fat Deposition

The deleterious impact of these specific lipotoxic molecules: ceramides, DAGs, and cholesterol, is not confined to isolated signaling pathways but manifests in a tissue‐specific manner, governed by the unique susceptibility and function of each organ. This ectopic lipid deposition creates distinct pathological landscapes in key metabolic tissues, where the molecular mechanisms of lipotoxicity converge to drive systemic metabolic failure. The liver, skeletal muscle, and pancreas emerge as a critical triad of organs whose coordinated dysfunction, fueled by ectopic fat, orchestrates the transition from IR to overt hyperglycemia.

#### 3.2.1 Hepatic Steatosis and IR

Ectopic fat deposition in the liver, manifesting as nonalcoholic fatty liver disease (NAFLD), is a cornerstone of hepatic metabolic dysfunction in T2D [121]. The accumulation of intracellular lipids is a primary instigator of hepatic IR [122]. This disrupts the downstream insulin signaling cascade [123]. A critical consequence is the failure of insulin to suppress hepatic glucose production that directly contributes to fasting hyperglycemia [124]. Furthermore, the insulin‐resistant state promotes de novo lipogenesis (DNL) through the activation of SREBP‐1c [124]. The combination of increased DNL and continued delivery of fatty acids from adipose tissue lipolysis overwhelms the liver's capacity for fat oxidation [125]. This hepatic VLDL overproduction is a major driver of the atherogenic dyslipidemia that profoundly increases cardiovascular risk in patients with T2D [47].

#### 3.2.2 Pancreatic Steatosis and β‐Cell Dysfunction

Fat accumulation in the pancreas, known as nonalcoholic fatty pancreas disease, directly impairs endocrine function through localized lipotoxicity and inflammation [126]. Unlike in the liver, pancreatic fat often involves the infiltration of adipocytes into the interlobular and peri‐islet spaces, creating a harmful paracrine environment [127]. These adipocytes release a constant stream of NEFAs and proinflammatory cytokines (e.g., TNF‐α, IL‐6) that bathe the nearby islets [3]. Within the β‐cell, chronic exposure to saturated fatty acids like palmitate promotes the formation of ceramides, which induce ERS and mitochondrial dysfunction, ultimately triggering apoptosis [128]. Furthermore, the lipid overload disrupts the finely tuned GSIS pathway [129]. It can uncouple mitochondrial metabolism from the triggering of exocytosis, leading to elevated basal insulin secretion but a blunted response to glucose [129]. The local inflammatory milieu also activates the NLRP3 inflammasome within islets, promoting caspase‐1‐mediated pyroptosis, a highly inflammatory form of cell death [130]. This combination of functional impairment and loss of β‐cell mass under the pressure of pancreatic steatosis is a critical factor in the progression from compensatory hyperinsulinemia to irreversible β‐cell failure and overt diabetes [131].

#### 3.2.3 Skeletal Muscle IR

Skeletal muscle, as the primary site for postprandial glucose disposal, becomes a major locus of metabolic dysfunction when it accumulates ectopic lipid [132]. Intramyocellular lipid, specifically in the form of DAG and ceramide, is a key mediator of this defect [133]. DAG accumulation activates novel PKCθ, which phosphorylates IRS‐1 on inhibitory serine residues [133]. This impedes the insulin signaling cascade, preventing the translocation of GLUT4 glucose transporters to the plasma membrane and severely reducing insulin‐mediated glucose uptake [134]. Simultaneously, the accumulation of ceramides exerts a parallel inhibitory effect by promoting the dephosphorylation of Akt via PP2A [135] and also contributes to mitochondrial dysfunction [136]. The result is a profound postprandial hyperglycemia, as ingested carbohydrates cannot be efficiently cleared into the muscle [137]. This muscle IR also has systemic repercussions; it diverts dietary carbohydrates toward the liver, where they serve as substrate for DNL, further exacerbating hepatic steatosis and dyslipidemia [138].

### 3.3 Proteostasis Imbalance: Islet Amyloid Polypeptide Toxicity and Impaired Autophagy

Beyond the direct impact of lipotoxicity, β‐cell failure in T2D is profoundly exacerbated by the toxic misfolding of islet amyloid polypeptide (IAPP) and a parallel decline in the proteostatic mechanisms designed to counteract such damage.

#### 3.3.1 IAPP Toxicity

The toxicity of hIAPP represents a unique and protein‐specific pathway to β‐cell failure. Cosecreted with insulin, hIAPP is prone to misfolding and forming toxic oligomers, particularly under the demanding conditions of insulin hypersecretion that characterize the compensatory phase of T2D [139]. Unlike the inert, extracellular amyloid fibrils found in late‐stage disease, these soluble oligomers are the primary cytotoxic species [140]. They exert their damaging effects through multiple mechanisms. They can directly integrate into the β‐cell plasma membrane, disrupting its integrity and inducing uncontrolled ion leakage [141]. Intracellularly, hIAPP oligomers impair mitochondrial function, reducing ATP production and increasing ROS [142]. Furthermore, the accumulation of misfolded hIAPP within the ER overwhelms its folding capacity, triggering severe and chronic ERS, which can culminate in apoptosis [143]. This process is also highly inflammatory, as hIAPP aggregates can activate the NLRP3 inflammasome, leading to the production of proinflammatory IL‐1β, thereby creating a vicious cycle of local inflammation and β‐cell damage.

#### 3.3.2 Impaired Autophagy

In parallel to the increased production of toxic proteins, the β‐cell's primary clearance mechanism, autophagy, becomes critically impaired in T2D, creating a perfect storm for proteostatic collapse. Autophagy is a lysosomal degradation pathway essential for the turnover of damaged organelles, such as dysfunctional mitochondria (mitophagy), and the clearance of aggregated proteins like oligomeric hIAPP [144]. Under diabetic conditions, characterized by chronic nutrient excess and glucolipotoxicity, the autophagic flux in β‐cells is significantly suppressed [145]. This impairment stems from the disruption of key signaling pathways, including the downregulation of the energy‐sensor AMPK (an activator of autophagy) and the aberrant activation of mTOR (a potent inhibitor of autophagy) [81]. Consequently, toxic protein oligomers and defective organelles accumulate to pathogenic levels. The failure of mitophagy leads to a population of ROS‐generating mitochondria, exacerbating oxidative stress [25]. Simultaneously, the inability to clear hIAPP oligomers allows them to persist and amplify their toxic effects on membranes and organelles [143]. This failure in cellular housekeeping accelerates β‐cell dysfunction and apoptosis, representing a critical failure in the defense against proteotoxicity [144].

### 3.4 The Gut–Metabolism Axis: Intestinal Barrier and Microbial Metabolites

Beyond intracellular stress pathways, the gut microbiota emerges as a critical environmental interface, where dietary components are metabolized into key signaling molecules that systemically influence host metabolism and insulin sensitivity.

#### 3.4.1 Short‐Chain Fatty Acids

Short‐chain fatty acids (SCFAs), primarily acetate, propionate, and butyrate, are produced by the bacterial fermentation of dietary fiber in the colon and serve as pivotal mediators of metabolic health. These metabolites exert pleiotropic beneficial effects through multiple mechanisms. Butyrate, in particular, is a primary energy source for colonocytes and helps maintain intestinal barrier integrity, thereby reducing the translocation of proinflammatory lipopolysaccharide and mitigating metabolic endotoxemia. Recent studies highlight that SCFAs (especially propionate and butyrate) ameliorate diabetic pathogenesis by improving insulin sensitivity and glucose homeostasis, though their clinical application is limited by pharmacokinetic challenges [146]. SCFAs also act as ligands for specific G‐protein‐coupled receptors (GPCRs), such as GPR41 (FFA3) and GPR43 (FFA2), which are therapeutic targets in immuno‐metabolic diseases [147]. Activation of these receptors on enteroendocrine L‐cells stimulates the secretion of GLP‐1, which enhances glucose‐dependent insulin secretion, suppresses glucagon release, and promotes satiety. Furthermore, SCFAs, especially acetate and propionate, can reach the liver and peripheral tissues via the portal circulation, where they inhibit HDACs, leading to epigenetic modifications that enhance insulin signaling and reduce hepatic gluconeogenesis [148]. Notably, pharmacological inhibition of adipocyte IRF3/AIG1 signaling, a pathway modulated by SCFAs, reverses obesity‐induced IR and restores glucose homeostasis [149].

Emerging evidence also underscores the role of SCFAs in regulating immune responses and inflammation, with serum and stool SCFA levels linked to chronic inflammatory conditions [150]. Through their anti‐inflammatory, hormonal, and epigenetic actions, SCFAs represent a crucial link between a fiber‐rich diet, a healthy gut microbiota, and improved systemic glucose homeostasis [150]. However, their therapeutic potential is constrained by low molecular weight, off‐target effects, and poor palatability, necessitating novel delivery strategies [146].

#### 3.4.2 Branched‐Chain Amino Acids

Elevated circulating levels of branched‐chain amino acids (BCAAs): leucine, isoleucine, and valine, are strongly associated with IR and T2D risk, a correlation observed since the 1960s [151]. The mechanism linking BCAAs to IR involves impaired BCAA catabolism, where pharmacological activation of branched‐chain α‐ketoacid dehydrogenase—the rate‐limiting enzyme of BCAA oxidation—can lower plasma BCAAs but interestingly fails to improve insulin sensitivity when activated specifically in skeletal muscle [151]. In individuals with T2D, the ability of insulin to suppress plasma BCAAs is significantly impaired compared with both lean individuals and those with obesity [152], though exercise training shows no corrective effect on this impairment [152]. At the molecular level, BCAA accumulation leads to mitochondrial dysfunction evidenced by incomplete fatty acid oxidation [153], while chronic mTORC1 pathway activation contributes to insulin signaling impairment through IRS‐1 serine phosphorylation [153]. Notably, brown adipose tissue (BAT) demonstrates a unique role in BCAA catabolism, where impaired mitochondrial BCAA transport in BAT specifically induces systemic IR without affecting energy balance [153]. Clinically, elevated BCAA levels show differential patterns in diabetes complications, being lower in diabetic cardiomyopathy patients compared with T2D patients without cardiac involvement [154], while positively correlating with diabetic kidney disease progression [155]. These findings collectively position BCAAs as important metabolic signatures linking multiple organ systems to IR pathogenesis.

#### 3.4.3 Secondary Bile Acids

Secondary bile acids, such as deoxycholic acid and lithocholic acid, are produced by the gut microbiota through the biotransformation of primary bile acids synthesized in the liver. These metabolites function as important signaling molecules by activating specific host nuclear and membrane receptors, primarily the farnesoid X receptor (FXR) and the G‐protein‐coupled bile acid receptor 1 (GPBAR1, also known as TGR5) [156]. The activation of these receptors has profound effects on glucose and energy metabolism [157]. TGR5 activation on enteroendocrine L‐cells stimulates GLP‐1 secretion, improving insulin sensitivity and pancreatic β‐cell function. This effect is mediated through microbial‐derived bile acids like ωMCA and HCA via TGR5‐dependent pathways [158]. In contrast, the role of FXR is more complex and tissue dependent. Intestinal FXR activation has been shown to improve glucose tolerance through the ileal bile acid–FXR–GLP‐1 pathway [159], potentially by modulating ceramide synthesis and the production of fibroblast growth factors. However, hepatic FXR activation primarily regulates bile acid homeostasis via the ileal FGF15/19 endocrine pathway and can suppress DNL [160].

The balance and composition of the bile acid pool, shaped by the gut microbiome, are therefore critical for metabolic regulation. Gut microbiota‐related bile acid metabolism–FXR/TGR5 axis significantly impacts gut barrier function and inflammatory responses [161]. Dysbiosis can alter this balance, leading to a bile acid profile that fails to optimally engage these receptors, thereby contributing to metabolic dysfunction [159].

In summary, the systemic manifestations of T2D are characterized by a self‐reinforcing cycle of tissue‐specific dysfunction. Ectopic lipid deposition, proteotoxicity from IAPP aggregation, and gut‐derived metabolic signals do not operate in isolation but converge across the liver, pancreas, and skeletal muscle. This convergence disrupts organ function through lipotoxicity, inflammatory activation, and impaired cellular quality control, collectively driving and sustaining the core metabolic imbalances of hyperglycemia and IR.

## 4 Evolution of Therapeutic Interventions for T2D

**FIGURE 5:**
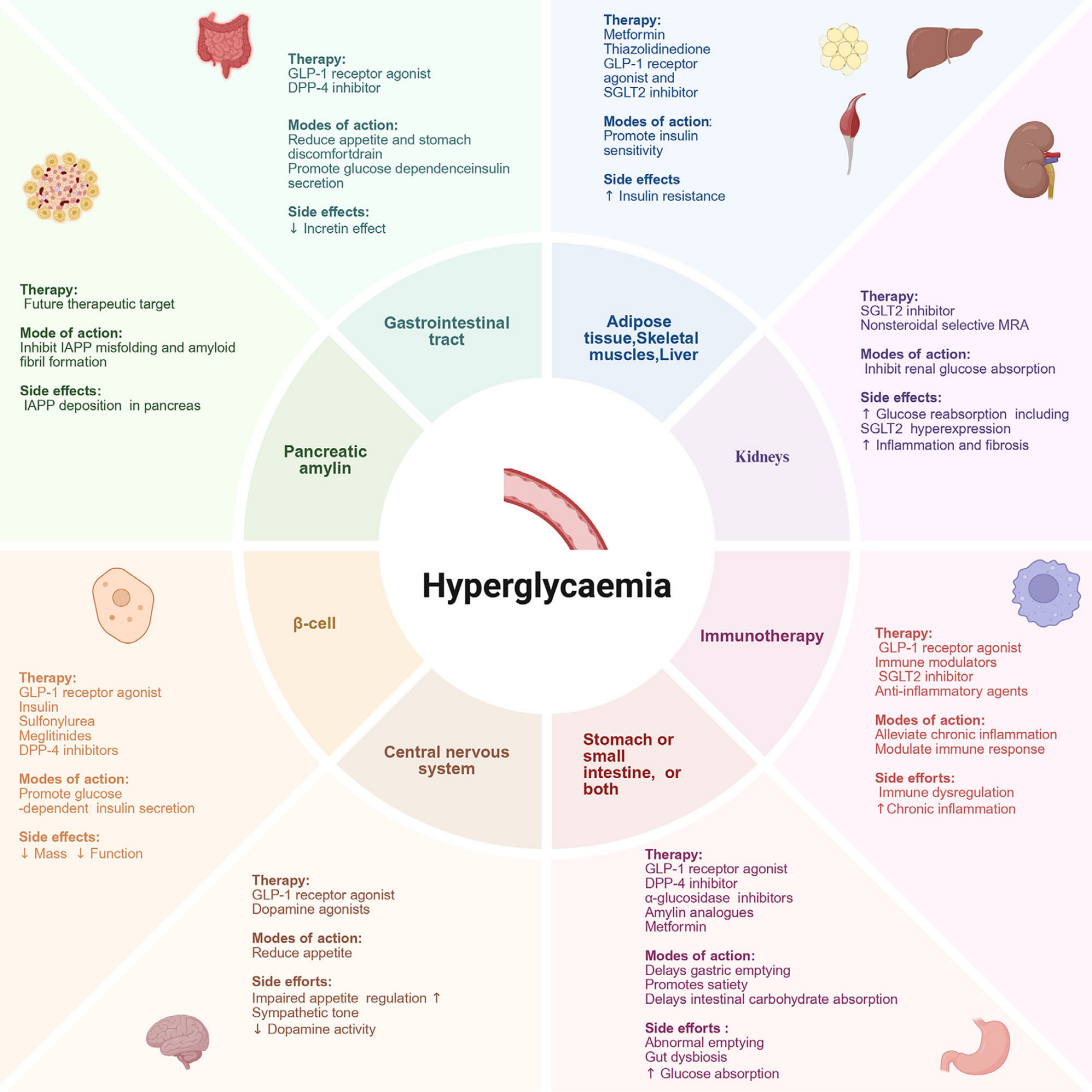
Summary of major therapeutic classes for T2D, their modes of action, and key side effects. The diagram illustrates various pharmacological agents used in T2D management, including SGLT2 inhibitors, metformin, thiazolidinediones, GLP‐1 receptor agonists, DPP‐4 inhibitors, insulin, sulfonylureas, meglitinides, α‐glucosidase inhibitors, amylin analogues, and emerging therapies. Modes of action include inhibiting renal glucose absorption, promoting insulin sensitivity, reducing appetite, promoting glucose‐dependent insulin secretion, inhibiting IAPP misfolding, alleviating chronic inflammation, modulating immune response, delaying gastric emptying, promoting satiety, and delaying intestinal carbohydrate absorption. Side effects include decreased glucose reabsorption, inflammation and fibrosis, insulin resistance, impaired appetite regulation, increased sympathetic tone, decreased dopamine activity, decreased β‐cell mass and function, IAPP deposition in pancreas, immune dysregulation, increased chronic inflammation, abnormal gastric emptying, gut dysbiosis, and increased glucose absorption. GLP‐1, glucagon‐like peptide‐1; DPP‐4, dipeptidyl peptidase‐4; IAPP, islet amyloid polypeptide.

The management of T2D has undergone a significant paradigm shift, evolving from a historically glucose‐centric approach which focused primarily on hemoglobin A1c (HbA1c) reduction through medications like metformin and insulin to a complications‐centric strategy that prioritizes the prevention of cardiovascular, renal, and other organ‐specific complications using agents such as SGLT2 inhibitors and GLP‐1 receptor agonists with proven cardio‐renal benefits [162]. This evolution reflects a deeper understanding of T2D as part of a broader MDS, leading to a current pathogenesis‐centric perspective that addresses underlying mechanisms like IR, inflammation, and ectopic lipid deposition through holistic interventions targeting obesity, dyslipidemia, and hypertension [120]. Consequently, the concept of MDS‐related target organ damage has emerged, emphasizing that complications arise from interconnected metabolic disorders rather than hyperglycemia alone, contrasting with the traditional diabetic complications framework and advocating for integrated management to improve overall patient outcomes and quality of life [163]. The mechanisms of action, along with the pivotal clinical trials that underpin the efficacy and organ‐protective benefits of the major pharmacological classes are discussed (Figure 5).

### 4.1 Foundational Nonpharmacological Interventions

While pharmacological advances offer powerful tools for managing T2D, foundational nonpharmacological interventions remain the cornerstone of disease management and prevention. These strategies target the root causes of metabolic dysfunction such as overnutrition, physical inactivity, and obesity, which provide the essential substrate upon which all other therapies build, forming the first‐line defense in the global effort to curb the T2D epidemic.

#### 4.1.1 Lifestyle Modifications

Foundational nonpharmacological interventions are essential in the management of T2D, focusing on lifestyle modifications that address the underlying metabolic dysfunctions and promote overall health. Medical nutrition therapy (MNT) plays a central role, with evidence‐based dietary patterns such as the Mediterranean diet, rich in monounsaturated fats, fruits, vegetables, and whole grains demonstrating significant improvements in glycemic control and cardiovascular health by reducing inflammation and oxidative stress [164]. Similarly, the dietary approaches to stop hypertension diet emphasizes low sodium intake and high consumption of potassium‐rich foods, aiding in blood pressure management and insulin sensitivity, while low‐carbohydrate diets (typically restricting carbohydrates to below 130 g/day) facilitate weight loss and HbA1c reduction by promoting ketogenesis and fat utilization [165]. These dietary strategies are tailored to individual preferences and comorbidities, ensuring adherence and sustainability through personalized planning that minimizes processed foods and added sugars. Complementing MNT, physical activity recommendations advocate for a combination of aerobic exercise (e.g., brisk walking, cycling, or swimming for at least 150 min per week at moderate intensity) and resistance training (2–3 sessions weekly targeting major muscle groups) to enhance insulin sensitivity, glucose uptake via GLUT4 translocation, and cardiorespiratory fitness, collectively lowering HbA1c by 0.5–1.0% and reducing cardiovascular risk [166]. Weight management strategies are integral, with a 5–10% loss of body weight proven to yield clinical benefits such as improved β‐cell function, reduced hepatic steatosis, and potential T2D remission, achieved through caloric restriction, behavioral coaching, and, when necessary, adjunctive therapies [165].

#### 4.1.2 Advanced Nonpharmacological Approaches

Advanced nonpharmacological approaches offer powerful interventions for T2D. Bariatric or metabolic surgery represents a potent option, with procedures working through multifaceted mechanisms including alterations in bile acid metabolism and modifications to the gut microbiome [167]. These changes lead to improvements in insulin sensitivity, as gut microbiome dysfunctions are associated with metabolic diseases through mechanisms like reduced microbial diversity and bile acid metabolism impairment [168]. The role of the gut microbiome is increasingly recognized, with interventions showing promise for glycemic improvements [169]. Diet and obesity contribute to IR and T2D in part via the gut microbiome, as gut‐derived metabolites are enriched in diabetes‐prone models. Restoring healthier gut microbiota composition may strengthen intestinal barrier integrity, as the gut microbiome's interaction with metabolites depends on diabetes status [170]. Certain antidiabetic drugs like metformin alter gut microbiome composition in T2D, though most studies have focused on fecal samples [171]. Metformin's protective effects may be associated with gut microbiome modulation, as microbial composition influences metabolic functions. Combined pharmacological approaches (e.g., metformin with liraglutide) also demonstrate gut microbial modulation effects in diabetes management [172]. For diabetes management, key interventions include tailored dietary and physical activity programs, as health behaviors play a key role in T2D development. However, evidence on specific nonpharmacological interventions’ impact remains limited, particularly regarding optimal approaches for metabolic health improvement [173].

#### 4.1.3 Art as a Catalyst: From Psychological Intervention to Cocreating a Health Ecosystem

In traditional diabetes management, psychological interventions such as cognitive‐behavioral therapy and mindfulness‐based stress reduction can alleviate disease‐related anxiety and enhance self‐efficacy, yet they are often constrained by their individualized and cognition‐oriented frameworks. Artistic interventions, with their unique capacity for empathy and experiential engagement, are reshaping this model. Psychologically modeled education might be the most beneficial way of promoting physical activity, and intervention setting and facilitator type should be considered when designing interventions for improving physical activity level in working‐age people with T2D [174]. They translate abstract health knowledge into visual narratives and interactive behaviors through embodied participation such as community murals depicting food systems or piano stairs that encourage physical activity [175]. These approaches not only reach cultural and age groups often excluded by digital tools but also strengthen community identity and a sense of health responsibility through collective creation. Art does not replace psychological therapies but serves as a bridge connecting individual behavioral change with systemic environments [175]. It integrates the cognitive restructuring of CBT and the awareness training of MBSR, yet does so in a low‐threshold, highly resonant manner, embedding health‐promoting behaviors into daily life and public spaces. The Women's Wellness with T2D Programme is an example of a gender‐specific intervention that fosters self‐efficacy, encouraging positive wellbeing behaviors to enhance diabetes outcomes [176]. This form of “experiential education” not only reduces psychological resistance but also empowers residents as active agents in health actions through cocreation, thereby driving sustainable change on both emotional and behavioral levels. Thus, art functions as a binding agent that integrates psychological mechanisms, environmental support, and community resources, advancing health management from an “individual treatment” model toward a transformative “cocreated ecosystem” [175]. With specific protocols, a variety of tailored approaches can induce T2D remission for patients with newly diagnosed T2D who are able to subscribe to strict protocols [177].

### 4.2 Pharmacological Agents: From Glycemic Control to Organ Protection

While foundational nonpharmacological interventions such as lifestyle modifications, metabolic surgery, and art‐based health promotion form the essential substrate for managing T2D, many patients require pharmacological support to achieve and maintain glycemic targets and prevent complications. The evolution of T2D therapeutics has expanded beyond traditional glucose‐lowering agents to include modern drugs with demonstrated cardio‐renal benefits and mechanisms targeting underlying pathophysiological processes. Notably, the CNS plays a role in the pathophysiology and treatment of T2D. It is a key regulator of energy homeostasis, appetite, satiety, and systemic metabolism [178]. Dysregulation within CNS circuits, particularly those involving hypothalamic and brainstem regions, can contribute to hyperphagia, weight gain, and impaired glucose control [179]. Consequently, several modern therapeutic classes, most notably GLP‐1 receptor agonists, exert their beneficial effects partly through direct action on CNS receptors, reducing appetite, promoting satiety, and modulating sympathetic tone [179, 180, 181], thereby addressing core metabolic dysfunctions from a central perspective.

#### 4.2.1 First‐Line and Traditional Agents

First‐line and traditional pharmacological agents remain foundational in T2D management, offering proven efficacy though with varying risk–benefit profiles that guide their contemporary use. Metformin, often initiated as first‐line therapy, primarily reduces hepatic gluconeogenesis through AMPK activation and mitochondrial complex I inhibition, leading to improved insulin sensitivity and modest HbA1c reductions of 1.0–1.5% with minimal hypoglycemia risk and weight neutrality. Animal studies demonstrate metformin's neuroprotective effects beyond glucose control [182], while clinical evidence supports its multiorgan effects including gut‐mediated glucose‐lowering mechanisms [183]. Its ongoing role persists due to low cost and extensive experience, though guidelines now prioritize agents with cardio‐renal benefits in high‐risk patients. Sulfonylureas (e.g., glimepiride) and meglitinides (e.g., repaglinide) act as insulin secretagogues by binding to sulfonylurea receptor 1 (SUR‐1) on pancreatic β‐cells, closing KATP channels to stimulate insulin secretion. Preclinical studies reveal differential cardiovascular risks associated with their affinity for cardiac mitoKATP channels [184], confirmed by clinical cohort studies showing high‐affinity sulfonylureas (glyburide/glipizide) combined with metformin increase major adverse cardiovascular events (MACEs) (HR 1.18) and severe hypoglycemia (HR 1.82) compared with low‐affinity agents (gliclazide/glimepiride) [184]. They provide high glucose‐lowering efficacy (HbA1c reduction up to 1.5%) but carry significant limitations including weight gain, high hypoglycemia risk (especially in elderly or renal impairment), and potential β‐cell exhaustion, with clinical data showing metformin–sulfonylurea combinations confer higher dementia risk than metformin–thiazolidinedione (TZD) regimens [185], relegating them to later‐line use. TZDs [186] (TZDs like pioglitazone) function as PPAR‐γ agonists, enhancing insulin sensitivity in adipose tissue, muscle, and liver by promoting adipocyte differentiation and fatty acid storage. Nationwide cohort studies demonstrate their cardiovascular safety profile compared with sulfonylureas when added to metformin [187], with real‐world evidence showing metformin–TZD combinations have lower dementia risk than metformin–sulfonylureas [185]. They result in HbA1c reductions of 0.5–1.4% with low hypoglycemia risk, and clinical trials associate them with reduced gastrointestinal cancer risk (HR 0.86 vs. other agents) [14]. Unique benefits include potential cardiovascular risk reduction and improved lipid profiles in some studies, but risks such as weight gain, fluid retention (particularly problematic in heart failure (HF) patients as shown in epidemiological studies [188]), and bone fractures necessitate careful patient selection and monitoring.

#### 4.2.2 Modern Agents With Cardio‐Renal Benefits

Modern agents with cardio‐renal benefits have revolutionized T2D management by shifting focus beyond glycemic control to direct organ protection. SGLT2 inhibitors mechanistically block sodium–glucose cotransporters in the renal proximal tubules, promoting glycosuria and natriuresis; this translates to robust cardiovascular outcomes including reduced HF hospitalizations (27–35% risk reduction in trials) [181, 189] and slowed renal disease progression (40% lower risk of worsening nephropathy in CREDENCE and DAPA‐CKD trials) [190] through hemodynamic and metabolic effects. GLP‐1 receptor agonists (e.g., semaglutide, dulaglutide) enhance glucose‐dependent insulin secretion, suppress glucagon, delay gastric emptying, and promote satiety via CNS receptors, yielding significant weight loss (5–10% body weight reduction) and cardiovascular risk reduction (14% MACEs risk reduction in meta‐analyses) [191] through antiatherosclerotic and anti‐inflammatory pathways. The dual GIP/GLP‐1 receptor agonist tirzepatide demonstrates superior efficacy by synergistically targeting both incretin receptors, achieving unprecedented HbA1c reductions (up to −2.4%) and weight loss (up to −11.7 kg in SURPASS trials) while showing promising cardio‐renal protective potential in ongoing outcomes trials [192], positioning it as a transformative option for comprehensive metabolic management. Clinical trials have demonstrated that both SGLT2 inhibitors and GLP‐1 receptor agonists significantly reduce MACEs compared with DPP‐4 inhibitors [193] or sulfonylureas [191], with meta‐analyses showing consistent benefits across different populations [194]. Combination therapy studies suggest additive effects, with GLP‐1 receptor agonists maintaining their 21% MACEs risk reduction regardless of concurrent SGLT2 inhibitor use [195]. These agents collectively underscore a paradigm where metabolic and organ‐protective benefits are integral to modern T2D therapeutics.

#### 4.2.3 Additional Therapeutic Classes

Additional therapeutic classes in T2D management provide tailored options for diverse patient needs, complementing first‐line agents with unique mechanisms and applications. DPP‐4 inhibitors, such as sitagliptin and linagliptin, exert their effects by blocking the degradation of endogenous incretin hormones (GLP‐1 and GIP), thereby enhancing glucose‐dependent insulin secretion and suppressing glucagon release; this results in modest HbA1c reductions (0.5–0.8%) with a low risk of hypoglycemia and weight neutrality, positioning them as suitable for older adults or those intolerant to more potent agents. Clinical trials demonstrate that while DPP‐4 inhibitors are well tolerated, their cardiovascular benefits are less pronounced compared with GLP‐1 receptor agonists, as shown in network meta‐analyses where SGLT2 inhibitors and GLP‐1 agonists reduced MACE more effectively than DPP‐4 inhibitors [191]. Preclinical studies also highlight their potential neuroprotective effects in Parkinson's disease models, linked to improved nigrostriatal dopamine function [196]. α‐Glucosidase inhibitors [197] (e.g., acarbose) target intestinal enzymes to delay carbohydrate digestion and absorption, primarily reducing postprandial hyperglycemia with HbA1c lowering of 0.5–0.8%; their niche application lies in managing meal‐related glucose spikes, particularly in populations with high carbohydrate intake. Clinical data reveal their mortality‐neutral profile in COVID‐19 patients with T2D [198], though limitations include frequent gastrointestinal adverse effects (e.g., flatulence and diarrhea) and minimal impact on fasting glucose, restricting use to adjunctive therapy. Observational studies note mixed evidence for anticancer effects, warranting further investigation [199].

Insulin therapy remains indispensable for advanced T2D, with regimens ranging from basal insulin [200] (e.g., glargine) for sustained control to prandial or premixed formulations for post‐meal coverage. Clinical studies underscore its association with increased mortality in COVID‐19 cohorts [198], prompting modern approaches to mitigate risks, such as combining basal insulin with GLP‐1 receptor agonists to reduce hypoglycemia and weight gain [201]. Preclinical research explores “smart insulin” formulations with glucose‐dependent action, while real‐world trials validate technologies like continuous glucose monitoring for personalized dosing [202]. Together, these classes highlight a nuanced arsenal addressing specific pathophysiological defects and clinical scenarios in T2D care, supported by animal models elucidating mechanistic insights [202] and comparative effectiveness trials guiding therapeutic selection [203, 204, 205]. The mechanisms of action, along with the pivotal clinical trials that underpin the efficacy and organ‐protective benefits of the major pharmacological classes discussed in this section—from first‐line agents to modern drugs—are systematically summarized in Table 2.

**TABLE 2:** Therapeutic interventions for T2D by pathway, mechanism, and key trials.

Therapeutic class	Representative agents	Mechanism of action	Targeted pathway/process	Key clinical trials/outcomes	References
Biguanides	Metformin	AMPK activation; inhibits mitochondrial complex I; reduces hepatic gluconeogenesis	AMPK pathway; mitochondrial function	UKPDS (long‐term cardiovascular risk reduction); improved insulin sensitivity	[182, 183]
SGLT2 inhibitors	Empagliflozin, dapagliflozin	Inhibits SGLT2 in renal proximal tubules → glycosuria, natriuresis	Renal glucose reabsorption; hemodynamic pathways	EMPA‐REG OUTCOME (CV benefit); DAPA‐CKD (renal protection); CREDENCE (nephropathy)	[181, 189, 190]
GLP‐1 receptor agonists	Semaglutide, liraglutide	Enhances glucose‐dependent insulin secretion; suppresses glucagon; delays gastric emptying; promotes satiety via CNS	Incretin pathway; CNS appetite regulation	LEADER, SUSTAIN‐6 (CV risk reduction); STEP (weight loss); LEAN (MASLD improvement)	[189, 191]
Dual GIP/GLP‐1 RA	Tirzepatide	Coagonism of GIP and GLP‐1 receptors → enhanced insulin secretion and satiety	Incretin system; energy homeostasis	SURPASS (superior HbA1c reduction & weight loss); SURMOUNT (obesity)	[212, 246]
DPP‐4 inhibitors	Sitagliptin, linagliptin	Inhibits DPP‐4 → increases endogenous GLP‐1 and GIP	Incretin pathway	TECOS (CV safety); low hypoglycemia risk; neuroprotective potential in preclinical models	[191, 193]
Thiazolidinediones (TZDs)	Pioglitazone	PPAR‐γ agonist → improves insulin sensitivity in adipose tissue, muscle, liver	PPAR‐γ pathway; adipokine regulation	PROactive (CV outcomes); PIVENS (MASLD improvement); reduced dementia risk with metformin combination	[185, 186, 187]
Sulfonylureas	Glimepiride, gliclazide	Binds SUR‐1 → closes KATP channels → membrane depolarization → stimulates insulin secretion	β‐Cell membrane depolarization	UKPDS (glycemic control); differential CV risk based on SUR affinity; higher hypoglycemia risk	[184, 185]
α‐Glucosidase inhibitors	Acarbose	Inhibits intestinal α‐glucosidase → delays carbohydrate absorption	Postprandial glucose modulation	STOP‐NIDDM (T2D prevention); ACE (CV outcomes neutral); reduces postprandial hyperglycemia	[197, 198]
Insulin therapy	Glargine, degludec	Exogenous insulin replacement → promotes glucose uptake and suppresses HGP	Insulin signaling pathway	ORIGIN (CV outcomes); basal‐bolus regimens for advanced T2D; modern combinations to reduce hypoglycemia	[200, 202]
Mito‐dynamics modulators (experimental)	Mdivi‐1	Inhibits Drp1 → reduces mitochondrial fission	Mitochondrial dynamics; oxidative stress	Preclinical models show improved β‐cell function; human trials pending	[20, 21, 22]
Mitophagy enhancers (experimental)	Urolithin A	Enhances mitophagy → clears dysfunctional mitochondria	PINK1–Parkin pathway; mitochondrial quality control	Preclinical models (improved mitochondrial function); clinical studies ongoing	[26, 27]
Anti‐inflammatory agents	Canakinumab (anti‐IL‐1β)	Inhibits IL‐1β → reduces inflammation and IR	NLRP3 inflammasome; inflammatory signaling	CANTOS (reduced CV events); T2D subgroup analysis; improved insulin sensitivity with metformin	[47, 48]
Bile acid modulators	Obeticholic acid (FXR agonist)	Activates FXR → modulates bile acid metabolism and glucose homeostasis	FXR/TGR5 signaling; gut–liver axis	FLINT (NASH improvement); glycemic benefits in T2D under investigation	[156, 157]
Mineralocorticoid receptor antagonists	Finerenone	Nonsteroidal MRA; reduces inflammation and fibrosis	Mineralocorticoid receptor pathway	FIDELIO‐DKD, FIGARO‐DKD (renal and CV benefits in T2D with CKD)	[223, 236]
Art‐based interventions	Community murals, piano stairs	Promotes psychological well‐being, community engagement, sustainable behavior change	Behavioral psychology; community health ecosystem	Pilot studies show improved adherence and mental health; integrated with lifestyle programs	[174, 175, 176, 177]

Abbreviations: ACE, acarbose cardiovascular evaluation; AMPK, AMP‐activated protein kinase; CANTOS, canakinumab anti‐inflammatory thrombosis outcomes study; CKD, chronic kidney disease; CNS, central nervous system; CREDENCE, canagliflozin and renal events in diabetes with established nephropathy clinical evaluation; CV, cardiovascular; DAPA‐CKD, dapagliflozin and prevention of adverse outcomes in chronic kidney disease; DPP‐4, dipeptidyl peptidase‐4; Drp1, dynamin‐related protein 1; EMPA‐REG OUTCOME, empagliflozin cardiovascular outcome event trial in T2D patients; FIDELIO‐DKD, finerenone in reducing kidney failure and disease progression in diabetic kidney disease; FIGARO‐DKD, finerenone in reducing cardiovascular mortality and morbidity in diabetic kidney disease; FXR, farnesoid X receptor; GIP, glucose‐dependent insulinotropic polypeptide; GLP‐1, glucagon‐like peptide‐1; HGP, hepatic glucose production; IL‐1β, interleukin‐1 beta; IR, insulin resistance; KATP, ATP‐sensitive potassium; LEADER, liraglutide effect and action in diabetes: evaluation of cardiovascular outcome results; LEAN, liraglutide efficacy and action in NASH; MASLD, metabolic dysfunction‐associated steatotic liver disease; Mdivi‐1, mitochondrial division inhibitor 1; MRA, mineralocorticoid receptor antagonist; NASH, nonalcoholic steatohepatitis; NLRP3, NLR family pyrin domain containing 3; ORIGIN, outcome reduction with initial glargine intervention; PINK1, PTEN‐induced putative kinase 1; PIVENS, pioglitazone versus vitamin E versus placebo for the treatment of nondiabetic patients with nonalcoholic steatohepatitis; PPAR‐γ, peroxisome proliferator‐activated receptor gamma; PROactive, prospective pioglitazone clinical trial in macrovascular events; RA, receptor agonist; SGLT2, sodium–glucose cotransporter 2; STEP, semaglutide treatment effect in people with obesity; SUR, sulfonylurea receptor; SURMOUNT, tirzepatide once‐weekly in the treatment of obesity; SURPASS, tirzepatide versus semaglutide once weekly in patients with T2D; SUSTAIN, semaglutide unabated sustainability in treatment of T2D; TECOS, trial evaluating cardiovascular outcomes with sitagliptin; TGR5, Takeda G protein‐coupled receptor 5; TZDs, thiazolidinediones; UKPDS, UK Prospective Diabetes Study.

### 4.3 Personalized and Complication‐Centric Management and Key Trials

Building upon the expanding arsenal of pharmacological agents with distinct mechanisms and clinical profiles, it becomes evident that effective management of T2D requires more than a one‐size‐fits‐all application of these therapies. The choice, sequence, and intensification of treatment must be strategically tailored to the individual patient's clinical characteristics, comorbidities, and risk factors.

#### 4.3.1 Patient‐Specific Considerations and Treatment Intensification

Patient‐specific considerations in T2D management are paramount for optimizing outcomes and minimizing risks, requiring a tailored approach that accounts for individual comorbidities, metabolic profiles, and vulnerabilities. Comorbidity‐based selection prioritizes agents with proven benefits for specific conditions; for instance, in patients with established atherosclerotic CVD (ASCVD), GLP‐1 receptor agonists and SGLT2 inhibitors are preferred due to their demonstrated reductions in MACEs, as shown in dedicated clinical trials evaluating cardiovascular and renal outcomes in T2D patients [206]. In HF or CKD, SGLT2 inhibitors take precedence for their robust effects on hospitalization risk and renal progression, supported by trials like DAPA‐CKD and DAPA‐HF, which included participants with and without T2D [207].

Weight status considerations further refine therapy, favoring GLP‐1 receptor agonists and SGLT2 inhibitors for weight loss (5–10% reduction) in obese patients, while preclinical studies highlight the role of metabolic dysregulation in diabetic complications, emphasizing the need for targeted therapies [208]. DPP‐4 inhibitors are preferred for weight neutrality in stable individuals, whereas agents like insulin or sulfonylureas (which promote weight gain) are avoided unless necessary.

Hypoglycemia risk stratification guides choices toward safer options such as metformin, DPP‐4 inhibitors, or SGLT2 inhibitors in vulnerable populations (e.g., elderly, renal impairment), as evidenced by clinical trials like Diaplate (NCT03460899), which investigated hypoglycemia's impact on platelet activation in T2D [209]. Continuous glucose monitoring integration enhances safety in these cases.

Age and frailty considerations in geriatric T2D management emphasize relaxed glycemic targets (HbA1c < 8.0%) and simplified regimens to avoid polypharmacy, aligning with findings from trials like ARMMS‐T2D, which compared bariatric surgery to medical/lifestyle management in T2D [210]. Agents with low hypoglycemia potential (e.g., linagliptin) are prioritized, alongside nonpharmacological support like nutritional counseling, as highlighted in translational research [211].

This holistic approach, informed by preclinical models of diabetes pathogenesis and clinical trials such as SURPASS‐1 (evaluating novel therapies in diet/exercise‐controlled T2D) [212], ensures precision medicine in T2D, balancing efficacy, safety, and individual needs. Future research should address gaps identified in trials, including the differentiation of T1D and T2D in datasets and the applicability of high‐income country findings to LMICs [213].

Treatment intensification strategies in T2D are critical for achieving glycemic targets and preventing complications, guided by systematic approaches that prioritize efficacy, safety, and individualization. Baseline HbA1c stratification serves as the cornerstone, with clinical trials like the GRADE study demonstrating the effectiveness of adding glucose‐lowering medications to metformin monotherapy in patients with HbA1c 6.8–8.5% and diabetes duration <10 years [214]. For those with HbA1c levels within 0.5% of their target, lifestyle interventions may suffice, as evidenced by clinical trials showing lifestyle modification programs can prevent or delay T2D progression [215]. Levels 0.5–1.5% above target warrant monotherapy with metformin or other first‐line agents, while elevations of 1.5–3.0% necessitate dual therapy using agents with complementary mechanisms.

Preclinical animal studies have revealed multiple pathophysiological defects in T2D, including β‐cell dysfunction and IR, supporting the rationale for combination therapies targeting different aspects of disease pathophysiology [92]. The combination therapy rationales emphasize synergistic actions, such as pairing insulin sensitizers with incretin‐based therapies, an approach supported by clinical trials showing GLP‐1 receptor agonists can provide better glycemic control while reducing complications [216]. Similarly, combining SGLT2 inhibitors with GLP‐1 RAs addresses multiple pathways, with preclinical studies suggesting these agents may target different metabolic diseases that could be exploited for T2D treatments [217]. For HbA1c >9–10% or symptomatic hyperglycemia, clinical trials like SURPASS‐1 have demonstrated the efficacy of immediate insulin initiation or triple therapy in patients inadequately controlled by diet and exercise alone [212]. The debate between sequential versus initial combination approaches is informed by trials such as VERIFY, with preclinical animal models providing insights into disease progression mechanisms that support early intensive treatment [218]. Recent clinical trials have also explored novel treatment intensification options showing benefits in bodyweight, insulin dose, and hypoglycemia rates [219], while preclinical studies continue to identify new targets that may lead to novel therapies addressing both treatment and prevention of T2D complications [220]. The continuous emergence of new therapies in clinical trials, along with preclinical evidence from animal studies, underscores the importance of personalized treatment intensification strategies that consider both immediate glycemic control and long‐term complication prevention.

#### 4.3.2 Management of Cardiovascular and Renal Complications

CVD management in T2D is paramount due to the high prevalence of ASCVD, HF, and associated mortality, necessitating a multifaceted approach that integrates pharmacotherapy, risk factor control, and personalized strategies. Preclinical studies in diabetic mouse models have elucidated mechanisms linking hyperglycemia to accelerated atherosclerosis, though clinical trials highlight that factors beyond glucose (e.g., inflammation, hemodynamic stress) dominate CVD risk [221]. Agents with proven cardiovascular benefits, such as SGLT2 inhibitors and GLP‐1 receptor agonists are cornerstone therapies; SGLT2 inhibitors reduce HF hospitalizations by 27–35% and slow renal disease progression through hemodynamic effects and glycosuria, as evidenced in trials like EMPA‐REG, DAPA‐HF, and FIGARO‐DKD (where finerenone, a mineralocorticoid antagonist, also showed HF benefits in albuminuric T2D patients) [222, 223]. GLP‐1 RAs lower MACE by approximately 14% via anti‐inflammatory and antiatherosclerotic mechanisms, demonstrated in trials such as LEADER and SUSTAIN‐6 [189, 224].

Blood pressure and lipid management integration is essential, with targets of <130/80 mmHg achieved through ACE inhibitors or ARBs for renal and cardiovascular protection, supported by SPRINT trial data showing intensive systolic control benefits in T2D [225]. Statin therapy (e.g., atorvastatin) reduces LDL cholesterol by 30–50% and mitigates inflammation, synergizing with SGLT2i/GLP‐1 RA effects [226]. Preclinical models of diabetic cardiomyopathy underscore the role of oxidative stress and fibrosis, informing clinical trials targeting cardiorenal pathways [227].

Antiplatelet therapy (e.g., low‐dose aspirin) is reserved for secondary prevention in ASCVD, balancing ischemic benefit against bleeding risk [228]. For primary prevention, shared decision‐making is emphasized, reflecting a holistic strategy prioritizing evidence‐based interventions [229]. Recent trials like SCORED and SOLOIST‐WHF further validate SGLT1/2 inhibition (sotagliflozin) in T2D with CVD or HF, while meta‐analyses confirm SGLT2i efficacy in high‐risk T2D populations [230].

SGLT2 inhibitors have demonstrated robust renal protective effects in both preclinical and clinical studies. In preclinical research, systematic reviews of rodent models of diabetes (105 studies) showed that SGLT2 inhibitors improved glycemic control and various aspects of renal function, including beneficial effects on lipid metabolism, blood pressure, glomerulosclerosis, renal tubular fibrosis, apoptosis, oxidative stress, and inflammation [231]. Notably, these nephroprotective effects were observed regardless of whether treatment was initiated at diabetes onset or later [231]. However, studies in db/db mice more frequently yielded negative results compared with other models [231].

Clinically, large cardiovascular and renal outcome trials have consistently shown the renal benefits of SGLT2 inhibitors in patients with T2D, chronic kidney disease, or HF [232] These trials demonstrated that SGLT2 inhibitors reduce the risk of kidney disease progression, cardiovascular mortality, and HF hospitalizations, with benefits extending across various patient subgroups, including those with and without T2D [232]. The renoprotective effects of SGLT2 inhibitors are thought to be mediated through multiple mechanisms, including attenuation of glomerular hyperfiltration, improvement in glomerular hemodynamics, and reduction of inflammation and oxidative stress [233]. For instance, in animal models of T2D, SGLT2 inhibitors were shown to modulate glomerular hemodynamic function, either alone or in combination with renin–angiotensin–aldosterone system inhibitors [234]. Transcriptional profiling in human and mouse studies further revealed that SGLT2 inhibitors restore metabolic perturbations in proximal tubular cells and reduce inflammatory signaling (e.g., mTORC1) [233]. GLP‐1RAs also exhibit renal protective properties, though their benefits are generally more modest compared with SGLT2 inhibitors [235, 236]. Preclinical studies in diabetic and nondiabetic CKD models (e.g., subtotal nephrectomized rats) demonstrated that GLP‐1‐based therapies exert glucose‐independent kidney‐protective effects by modulating nutrient transport, redox sensing, and inflammation resolution [235]. Clinically, cardiovascular outcome trials (e.g., LEADER, REWIND, SUSTAIN‐6) reported a 15–36% risk reduction in albuminuria progression and composite renal events with GLP‐1 RAs, attributed to their anti‐inflammatory, antifibrotic, and blood pressure‐lowering properties [236].

Emerging therapies like finerenone, a nonsteroidal mineralocorticoid receptor antagonist, have shown additional renal and cardiovascular benefits in T2D patients with albuminuric CKD, as evidenced by the FIDELIO‐DKD and FIGARO‐DKD trials [236]. These trials highlighted finerenone's role in targeting oxidative stress and fibrosis, further expanding the therapeutic options for diabetic kidney disease.

#### 4.3.3 Management of Other Complications (Neuropathy, Retinopathy, Foot Ulcers)

Management of other complications in T2D requires a multifaceted approach. For diabetic neuropathy, pathogenesis‐targeted strategies include stringent glycemic control, supported by clinical evidence showing that adherence to a healthy lifestyle post‐diagnosis reduces neuropathy risk (RR 0.67, 95% CI 0.51–0.88) [237]. Preclinical studies highlight the role of oxidative stress mediated by Nox family NADPH oxidases in microvascular complications, including neuropathy [238]. Symptomatic management with gabapentinoids or duloxetine is complemented by photobiomodulation (PBM) therapy, which clinical trials demonstrate as effective for peripheral neuropathy and DFU pain [239].

In diabetic retinopathy, anti‐VEGF therapies and laser interventions are bolstered by systemic findings: serum magnesium levels are inversely associated with retinopathy risk (RR 0.77, 95% CI 0.61–0.98) [240], while vitamin D sufficiency lowers microvascular complication risks. Preclinical models emphasize inflammation's role in retinopathy pathogenesis, suggesting therapeutic targeting of inflammatory pathways [241].

For diabetic foot ulcers (DFUs), multidisciplinary care aligns with clinical data showing that DFUs have a mortality rate of 231 deaths per 1000 person‐years. Advanced wound therapies like sulfated hyaluronic acid/collagen nanofibrous skins show promise in preclinical diabetic mouse models, enhancing wound healing [242]. Clinical trials support PBM for ulcer‐related pain [239], while serum magnesium and glycemic control (HbA1c‐mediated) are linked to reduced foot complication risks [240].

#### 4.3.4 Special Populations (T2D With Obesity/MASLD, Elderly, Renal/Hepatic Impairment)

The management of T2D with comorbid obesity and metabolic dysfunction‐associated steatotic liver disease (MASLD) requires a targeted approach that addresses weight reduction and hepatic metabolic health, leveraging agents with dual benefits on glycemia and body composition. For weight loss, GLP‐1 RAs are highly effective, promoting satiety and reducing calorie intake through CNS actions. In preclinical studies, GLP‐1RAs demonstrated hepatic benefits by reducing liver fat accumulation and inflammation in diet‐induced obese mouse models [243]. Clinical trials like STEP and SCALE showed these agents lead to 5–10% body weight reduction and improved insulin sensitivity. The LEAN trial specifically demonstrated liraglutide's ability to reduce liver fat content and ALT levels in MASLD patients [244].

SGLT2 inhibitors contribute to modest weight loss (2–3 kg) via glycosuria and calorie excretion, while also reducing hepatic fat content through improved glucotoxicity and lipid metabolism. Though their effects are less pronounced than GLP‐1RAs, combination therapy studies show SGLT2i plus GLP‐1RA provides superior outcomes for MASLD compared with monotherapy [245].

Tirzepatide, a dual GIP/GLP‐1 receptor agonist, demonstrates superior efficacy with up to 15–20% weight loss in SURPASS and SURMOUNT trials, outperforming other agents by synergistically enhancing insulin secretion and suppressing appetite. This makes it a transformative option for severe obesity and metabolic syndrome [246]. For MASLD‐specific considerations, pioglitazone, a PPAR‐γ agonist, remains a cornerstone therapy by reducing hepatic steatosis and inflammation through adiponectin‐mediated improvements in insulin sensitivity and fatty acid oxidation, as shown in PIVENS and ACT NOW trials [247]. However, its use is limited by weight gain and fluid retention risks. GLP‐1RAs show particular promise in MASLD treatment. Phase 2 trials have demonstrated their beneficial effects on liver histology [248], while observational studies suggest they may reduce the long‐term risk of major adverse liver‐related outcomes [248]. The target trial emulation studies by further support that GLP‐1RA decreases risks of both liver and nonliver complications in T2D patients with MASLD.

These findings underscore a personalized strategy where combination therapies such as GLP‐1 RAs with pioglitazone or SGLT2 inhibitors, can be optimized based on individual patient profiles and treatment goals. The growing body of evidence from both preclinical models and clinical trials [245, 248] supports the use of these agents for managing the complex interplay between T2D, obesity and MASLD.

Management of T2D in elderly patients necessitates a nuanced, safety‐focused approach that prioritizes individualized care. Preclinical studies using rodent models with genetic mutations or nutrient metabolism manipulations have been instrumental in understanding glucose dysregulation and drug mechanisms, though their translational limitations for aging‐related physiology are acknowledged. Clinically, the ARMMS‐T2D trial demonstrated the long‐term glycemic benefits and safety of metabolic surgery versus medical/lifestyle interventions in T2D [210], though its applicability to elderly populations requires careful consideration of frailty and surgical risks. For hypoglycemia risk minimization, agents like DPP‐4 inhibitors, SGLT2 inhibitors, or GLP‐1 receptor agonists are preferred, supported by randomized trials showing their efficacy in HbA1c reduction without significant hypoglycemia [249]. Automated insulin delivery systems, validated in a 13‐week multicenter trial for insulin‐treated T2D, offer promise but require further study in older adults with functional limitations [250]. Continuous glucose monitoring and education are critical, as severe hypoglycemia can precipitate falls or cognitive decline.

Functional status and comorbidity management should involve geriatric assessments. The UK Prospective Diabetes Study legacy data highlighted enduring benefits of metformin in T2D, but its use in elderly patients with renal impairment necessitates dose adjustments [251]. SGLT2 inhibitors have shown cardiovascular and renal benefits in trials across disease states (HF, CKD), though estimated glomerular filtration rate (eGFR) thresholds must be observed [252]. Notably, 70% of T2D patients have NAFLD, with advanced fibrosis rates higher than the general population, underscoring the need for hepatic monitoring [253]. Recent consensus recommendations (2023) emphasize deprescribing and de‐intensification in older adults, advocating for relaxed targets (e.g., HbA1c <7.5‐8.0%) to avoid overtreatment [254]. Polypharmacy reduction strategies, such as stopping nonessential medications, are supported by observational studies and expert reviews [254].

The management of T2D in patients with renal or hepatic impairment requires meticulous dose adjustments and contraindication awareness. Preclinical studies in high‐fat diet/streptozotocin‐induced Type 2 diabetic mice demonstrated that mitochondrial impairment and lipid accumulation in heart tissues were attenuated by USP28 overexpression, suggesting potential organ‐protective mechanisms that may inform therapeutic strategies [255]. For renal impairment, dose modifications are stratified by eGFR. A meta‐analysis of 58,816 participants from SGLT2 inhibitor trials showed significantly lower kidney disease progression rates (33 vs. 48 events per 1000 patient‐years) compared with placebo, particularly in those with baseline albuminuria [256]. Metformin is contraindicated at eGFR <30 mL/min/1.73 m^2^ due to lactic acidosis risk, while SGLT2 inhibitors can be initiated down to eGFR ≥20 mL/min/1.73 m^2^ for cardio‐renal benefits, though clinical trials note reduced glucose‐lowering efficacy below eGFR 45 mL/min/1.73 m^2^, with HbA1c reduction differences observed between sitagliptin and canagliflozin in this subgroup [257].

DPP‐4 inhibitors such as linagliptin require no dose adjustment in renal failure, supported by a Phase 2 open‐label study in patients with chronic hepatitis B and renal impairment (eGFR 15–59 mL/min), which confirmed the safety profile of antiviral agents in this population [258]. Sulfonylureas necessitate dose reduction in advanced CKD (eGFR <30 mL/min/1.73 m^2^) to mitigate hypoglycemia risks, whereas GLP‐1 receptor agonists demonstrated weight loss >5% in most T2D patients in clinical trials, though exenatide is not recommended in severe CKD.

In hepatic impairment, a meta‐analysis of 2016 NAFLD patients (736 with T2D) revealed that T2D independently increased hepatic decompensation risk (sHR 2.15), highlighting the need for cautious drug selection [259]. Linagliptin and dulaglutide are preferred due to nonhepatotoxic profiles, while clinical studies in chronic hepatitis B patients with hepatic impairment confirmed the safety of switching to tenofovir alafenamide, providing a parallel for metabolic drug safety assessments [258]. TZDs are avoided in liver disease due to hepatotoxicity concerns, consistent with precision medicine trial findings that BMI >30 kg/m^2^ patients had differential glucose responses to these agents [257]. The CREDENCE trial (*n* = 2627) further validated biomarkers for renal/cardiac risk stratification in T2D with albuminuria, supporting personalized regimens [260]. Regular monitoring of eGFR and liver enzymes remains critical, as longitudinal cohort data showed hepatic decompensation occurred over a median 2.8 years in NAFLD‐T2D patients [259].

In summary, the evolution of T2D therapy marks a paradigm shift from a glucocentric model to a comprehensive strategy that prioritizes the prevention of cardio‐renal complications and addresses underlying pathogenesis. The introduction of SGLT2 inhibitors and GLP‐1 receptor agonists, with their proven organ‐protective benefits, alongside the refinement of lifestyle interventions and the innovative use of art‐based community engagement, has equipped clinicians with a multifaceted arsenal. This progress underscores the current era of personalized, complication‐centric management, where treatment selection is strategically tailored to the individual's comorbid conditions and risk profile, ultimately aiming to improve both longevity and quality of life for patients.

## 5 Conclusion and Prospects

This article provides a systematic review of the complex molecular pathogenesis and evolving therapeutic strategies for T2D. We elucidate that T2D is far from a mere disorder of glucose homeostasis; rather, it constitutes a self‐perpetuating vicious cycle arising from the convergence of dysfunctions across multiple organs including skeletal muscle, liver, adipose tissue, pancreas, kidneys, and brain. Core pathophysiological features encompass: dysregulation of the immunometabolic axis centered on macrophage polarization; disruption of mitochondrial dynamics and quality control in pancreatic β‐cells; lipotoxicity mediated by specific lipid molecules such as ceramides; and proteostasis imbalance due to toxic aggregation of IAPP and impaired autophagy. These mechanisms are intricately interwoven, collectively driving the critical transition from IR to overt hyperglycemia. The current treatment paradigm has shifted from a purely “glucocentric” approach to a complication‐centric “organ protection” model, and further toward an “etiology‐centric” strategy targeting the fundamental pathogenesis described above. Modern agents like SGLT2 inhibitors and GLP‐1 receptor agonists exemplify this shift, offering significant cardiovascular and renal benefits through multifaceted mechanisms that extend beyond glucose‐lowering.

Despite substantial advancements in our understanding of T2D, numerous challenges persist. First, current knowledge of processes such as macrophage heterogeneity and β‐cell dedifferentiation largely stems from animal models or in vitro studies, necessitating further validation of their precise roles and temporal dynamics in the human T2D progression. Second, the hierarchical relationships and crosstalk networks among different signaling pathways (e.g., inflammation, ERS, oxidative stress) are exceedingly complex; identifying core nodal points rather than peripheral pathways is crucial for developing effective targeted therapies. Additionally, while existing treatment strategies can effectively manage the condition, achieving T2D “remission” or “reversal” remains challenging, particularly in restoring the function and identity of β‐cells chronically exposed to glucolipotoxic environments, where tools are still limited.

Looking ahead, several directions hold promise. First, the application of multiomics technologies such as single‐cell sequencing, spatial transcriptomics, proteomics, and metabolomics to comprehensively analyze samples from T2D patients at different disease stages and with diverse genetic backgrounds may unveil novel disease endotypes and biomarkers, laying the groundwork for precise subtyping. Second, developing next‐generation precision therapies targeting identified key mechanisms is essential. For instance, correcting excessive mitochondrial fission through modulation of the Miro1–Drp1 axis or using drugs like Mdivi‐1; clearing toxic IAPP oligomers and dysfunctional mitochondria by enhancing autophagic flux or employing compounds such as urolithin A; and advancing regenerative medicine strategies aimed at reversing β‐cell dedifferentiation and promoting redifferentiation.

Ultimately, the prevention and management of T2D will evolve toward a highly personalized, preventive, and integrated new model. This entails combining genetic risk scores, multiomics profiles, and clinical data to construct predictive models using artificial intelligence algorithms, enabling intervention at early or even predisease stages. Simultaneously, the artistic promotion of nonpharmacological interventions (e.g., TRE) and the cocreation of community health ecosystems will integrate with novel drugs, cell therapies, and other advanced modalities, forming a comprehensive management network spanning from molecular to societal levels. Art‐based interventions emerge as a transformative force in diabetes care, effectively reducing psychological barriers, fostering community engagement, and promoting sustainable lifestyle changes through creative expression, thereby enhancing overall patient well‐being and treatment adherence. By continuously deepening our understanding of the disease essence and innovating intervention strategies, we aspire to ultimately break the vicious cycle of T2D and bring genuine health turnaround to hundreds of millions of patients worldwide.

## Author Contributions

S.N.F., X.Y.X., L.Z., J.Z., and L.W.C.: conceptualization and design. J.H, Q.Q.X, C.T.H., and J.C.: writing—data collection and analysis. S.J.Y.: writing – review and editing. All authors have read and agreed to the published version of the manuscript.

## Funding

This study is supported by grants from the National Natural Scientific Foundation of China (grant number 81670747), Hubei University of Traditional Chinese Medicine‐Huangshi Maternal and Child Health Hospital Joint Fund (2024ZXHSFY13), Hubei Provincial Natural Science Foundation Joint Project (2026AFC0013), Huangshi Municipal Health Commission Key Projects (WJ2024006), Huangshi‐Hengrui Pharmaceutical Joint Innovation Special Project (KJ2025027) to Sijun Yang; Yunnan Provincial Key Laboratory of Digital Protection and Intelligent Development of Ethnic Cultural Resources (2024KYPT604) to Jian Zhang.

## Ethics Statement

The authors have nothing to report.

## Conflicts of Interest

The authors declare no conflicts of interest.

## Data Availability

The authors have nothing to report.
