# Correction to “Type 2 Diabetes Mellitus: Molecular Pathogenesis and Therapeutic Interventions”

**DOI:** 10.1002/mco2.70968

**Published:** 2026-10-06

S. Fei, X. Xiong, L. Zhang, et al. “Type 2 Diabetes Mellitus: Molecular Pathogenesis and Therapeutic Interventions”, MedComm. 2026. https://doi.org/10.1002/mco2.70895.

Figure 4 (Progressive mechanisms of insulin resistance, lipotoxicity, inflammation, and β‐cell failure in T2D) contained an error in the pathway layout and labeling. Specifically, some arrows and molecular interactions were inaccurately depicted, which could mislead readers regarding the sequential progression of pathogenic events.

We have included a corrected version of the figure as follows, which accurately represents the integration of insulin resistance, lipotoxicity, inflammatory signaling, mitochondrial dysfunction, ER stress, and β‐cell failure as described in the text. We respectfully request that the original Figure 4 be replaced with this new file in the final published article.


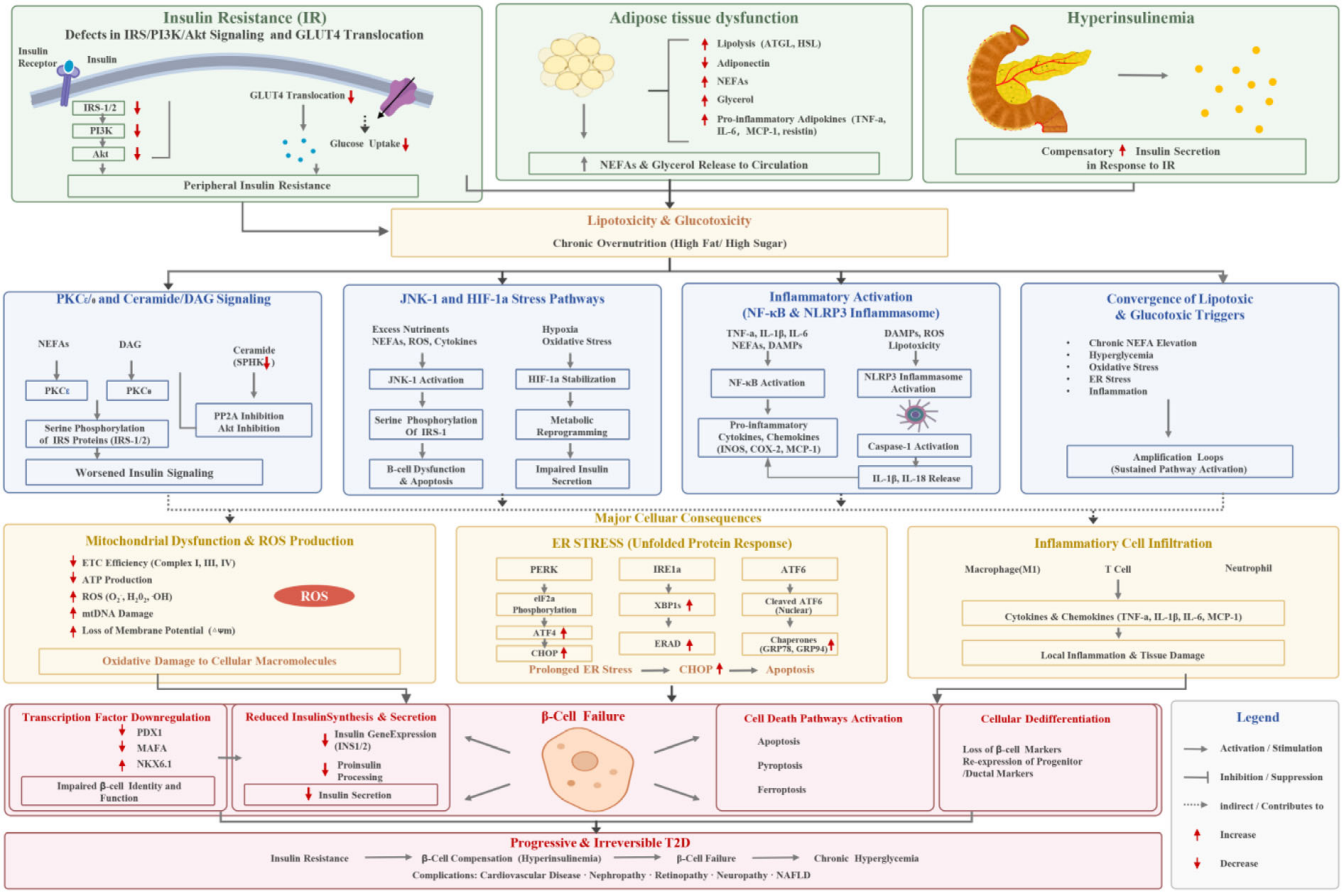


We apologize for this error and any confusion it may have caused. No blame is assigned to any individual; we are committed to maintaining the integrity of the scientific record.

